# Understanding the emergence of modern humans and the disappearance of Neanderthals: Insights from Kaldar Cave (Khorramabad Valley, Western Iran)

**DOI:** 10.1038/srep43460

**Published:** 2017-03-02

**Authors:** Behrouz Bazgir, Andreu Ollé, Laxmi Tumung, Lorena Becerra-Valdivia, Katerina Douka, Thomas Higham, Jan van der Made, Andrea Picin, Palmira Saladié, Juan Manuel López-García, Hugues-Alexandre Blain, Ethel Allué, Mónica Fernández-García, Iván Rey-Rodríguez, Diego Arceredillo, Faranak Bahrololoumi, Moloudsadat Azimi, Marcel Otte, Eudald Carbonell

**Affiliations:** 1 Institut Català de Paleoecologia Humana i Evolució Social (IPHES), Zona educacional 4, Campus Sescelades URV (Edif. W3), 43007 Tarragona, Spain; 2 Àrea de Prehistòria, Universitat Rovira i Virgili. Fac. de Lletres, Avinguda Catalunya 35, 43002 Tarragona, Spain; 3 Oxford Radiocarbon Accelerator Unit, Research Laboratory for Archaeology and the History of Art, University of Oxford, Dyson Perrins Building, South Parks Road, OX1 3QY Oxford, United Kingdom; 4 CSIC, Museo Nacional de Ciencias Naturales, c. José Gutiérrez Abascal 2, 28006 Madrid, Spain; 5 Bereich für Ur- und Frühgeschichtliche Archäologie, Friedrich Schiller Universität Jena, Löbdergraben 24a, Jena, 07743 Germany; 6 Neanderthal Museum, Talstrasse 300, D40822, Mettmann, Germany; 7 GQP-CG, Grupo Quaternario e Pre Historia do Centro de Geociencias (uI&D 73 e FCT), Portugal; 8 Unit Associated to the Centro Superior de Investigaciones Científicas (CSIC), 28006, Madrid, Spain; 9 Sezione di Scienze Preistoriche e Antropologiche, Dipartimento di Studi Umanistici, Università degli Studi di Ferrara (UNIFE), C. so Ercole I d’Este 32, 44121, Ferrara, Italy; 10 Facultad de Humanidades y Ciencias Sociales, Universidad Internacional Isabel I de Castilla, c. Fernán González 76, 09003, Burgos, Spain; 11 Iran’s Research Institute for Cultural Heritage and Tourism, Emam’s square, 11369-13431, Tehran, Iran; 12 University of Liège, Service of Prehistory, place du 20-Août 7, A1, 4000 Liège, Belgium

## Abstract

Kaldar Cave is a key archaeological site that provides evidence of the Middle to Upper Palaeolithic transition in Iran. Excavations at the site in 2014–2015 led to the discovery of cultural remains generally associated with anatomically modern humans (AMHs) and evidence of a probable Neanderthal-made industry in the basal layers. Attempts have been made to establish a chronology for the site. These include four thermoluminescence (TL) dates for Layer 4, ranging from 23,100 ± 3300 to 29,400 ± 2300 BP, and three AMS radiocarbon dates from charcoal samples belonging to the lower part of the same layer, yielding ages of 38,650–36,750 cal BP, 44,200–42,350 cal BP, and 54,400–46,050 cal BP (all at the 95.4% confidence level). Kaldar Cave is the first well-stratified Late Palaeolithic locality to be excavated in the Zagros which is one of the earliest sites with cultural materials attributed to early AMHs in western Asia. It also offers an opportunity to study the technological differences between the Mousterian and the first Upper Palaeolithic lithic technologies as well as the human behaviour in the region. In this study, we present a detailed description of the newly excavated stratigraphy, quantified results from the lithic assemblages, preliminary faunal remains analyses, geochronologic data, taphonomic aspects, and an interpretation of the regional paleoenvironment.

Understanding the initial spread of anatomically modern humans (AMHs) out of Africa is a key goal for palaeoanthropologists. AMHs originated in Africa and spread across the Middle East into Eurasia and towards Australia and the Americas. These AMHs were the first humans to occupy the latter two continents, but they replaced other populations in Eurasia. Because few well-dated human remains are available to study this dispersal process, the spread of AMHs is best documented by the appearance of the early phase of their culture, inferred to be the Aurignacian (but see ref. 1). In western Eurasia, this technocomplex replaced the Mousterian, associated in this region with Neanderthals. This transition may have occurred at approximately 50 to 40 ka (see refs 2, 3, 4, 5, 6, 7).

One key area relevant to the dispersal process is Iran and Iraq, particularly the Zagros Mountains. Since the first survey of the Zagros by D. Garrod in 1930, Palaeolithic deposits and surface finds have been reported from a large number of caves, rockshelters, and open-air sites, but few of them have been fully excavated. Locally, the early phase of the technocomplex associated with early AMHs is known as the Baradostian. The early Upper Palaeolithic assemblages are also known as the Rostamian, which is defined as a bladelet-based technocomplex^8^,^9^,^10^,^11^. Although Conard et al. view the Rostamian as an industry distinct from the Baradostian^11^, both terms refer to the early Upper Palaeolithic in the Zagros region.

Many of the researchers who study materials from the Zagros agree that the lithic assemblages from this region share some features with assemblages from central Europe and the Levant. These include typo-technological characteristics of the Aurignacian tradition as well as inter-assemblage variability^12^,^13^,^14^. Olszewski and Dibble^12^, for example, proposed changing the name of the Baradostian to the ‘Zagros Aurignacian’ in light of the perceived similarities with Aurignacian material.

There is disagreement, however, regarding whether the Upper Palaeolithic evolved from earlier Mousterian industries in the region^15^. Some authors have proposed that the Baradostian might have developed locally from the Mousterian^5^,^12^,^13^,^14^,^16^,^17^,^18^,^19^,^20^,^21^,^22^,^23^,^24^,^25^,^26^,^27^,^28^,^29^,^30^,^31^,^32^. On the one hand, recent work on two stratified assemblages from Warwasi and Yafteh support an *in situ* evolution of the Upper Palaeolithic from the local Mousterian^15^. That study, however, focused on only two assemblages; thus, the conclusions might not be fully applicable to the entire Zagros region. On the other hand, Tsanova^15^ raised doubts about whether the Iranian Zagros was the source of bladelet technology. However, the discovery of over 90 sites in the southern Zagros mostly associated with bladelet-based technologies—one of which dates to 40,000 cal. BP—suggests that the technology in the region featured a high degree of complexity^5^,^8^,^9^,^10^,^11^.

Additionally, the Zagros is more than 2,000 km long from northwest to southeast and up to several hundred kilometres wide from east to west^27^. Due to the lack of extensive surveys and archaeological excavations in the region, many aspects remain poorly understood. The latest typo-technological analyses on the lithic assemblage from the site of Ghar-e-Khar, for example, indicate the presence of multiple sites containing both Middle and Upper Palaeolithic sequences in the Zagros region. These findings confirm the potential for continued research into the Middle to Upper Palaeolithic transition^33^. However, only a few excavated sites contain uninterrupted archaeological sequences that include both Middle and Upper Palaeolithic deposits^34^. Some well-excavated sites, e.g., Yafteh, do not have Middle Palaeolithic occupation levels. Besides the reported sites in the Gilvaran and Ghamari caves^35^, Warwasi and Ghar-e-Khar are the only sites in the Iranian Zagros containing cultural remains belonging to both the Middle and Upper Palaeolithic (Fig. 1). To date, however, neither has been dated. Warwasi and Ghar-e-Khar were coarsely excavated (20 cm spits in Warwasi and 10–30 cm spits in Ghar-e-Khar). Chronometric control of the sites has been hampered by the poor preservation of organic material extracted from the archaeological sites and by political challenges and instability, which have made excavation work virtually impossible for more than 20 years. Here, we present the recently excavated and dated well-stratified sequence of Kaldar Cave, which documents the transition from the Middle to the Upper Palaeolithic.

## Results

### Site stratigraphy

Kaldar Cave is situated in the northern Khorramabad Valley at 48° 17′35″E longitude, 33°33′25″N latitude, and an elevation of 1,290 m above sea level. It is 16 m long, 17 m wide, and 7 m high. The potential of this site for excavation was first realized during a survey in 2010, when we started our regional study of the Khorramabad Valley as a goal-oriented research project. The first excavation^35^ was conducted in 2011–12.

The 2014–15 excavation focused on gaining a better understanding of the stratigraphy and obtaining samples for dating. We opened a 3 × 3 m trench near the entrance and kept a 50 cm bulk sample from the previous test pit (squares E5, E6, E7, F5, F6, F7, G5, G6 and G7) (Supplementary Fig. S1). The excavation was conducted using spits of 5 cm within each archaeostratigraphic unit, as well as 3D recording of all findings.

The excavated trench exposed an approximately 2-m (195-cm) section of the sedimentary deposit, which is characterized by five main layers. During fieldwork, distinctions within the layers were made according to minor sedimentological differences. Ongoing microstratigraphic research will provide a proper characterization of the sub-layers.

Layers 1 to 3 (including sub-layers 4 and 4II) consist of ashy sediment with a blackish-green colour containing both thick and thin angular limestone clasts. These layers varied in thickness from 60 to 90 cm and contained many phases dating to the Holocene: the Islamic and historical eras, Iron Age, Bronze Age, Chalcolithic, and Neolithic. However, due to the presence of some bioturbation in these layers, the phases were recognized only by a preliminary study of the potsherds, metal artefacts and some diagnostic lithic artefacts from the lower layers.

Layer 4 (including sub-layers 5, 5II, 6 and 6II) consists a silty but compact dark-brown sediment with cultural remains from the Upper and early Upper Palaeolithic. In the uppermost parts of this layer, two fireplaces made of clay were recovered and dated through thermoluminescence, yielding ages that ranged from 23100 ± 3300 to 29400 ± 2300 BP (Table 1). The dates obtained show that these fireplaces were made or re-used from existing older sediment from the upper part of this layer in the later stages of the Upper Palaeolithic. AMS radiocarbon dates of 38650–36750 cal BP, 44200–42350 cal BP, and 54400–46050 cal BP have been obtained from charcoal material located below this layer (Table 2).

**Figure 2:**
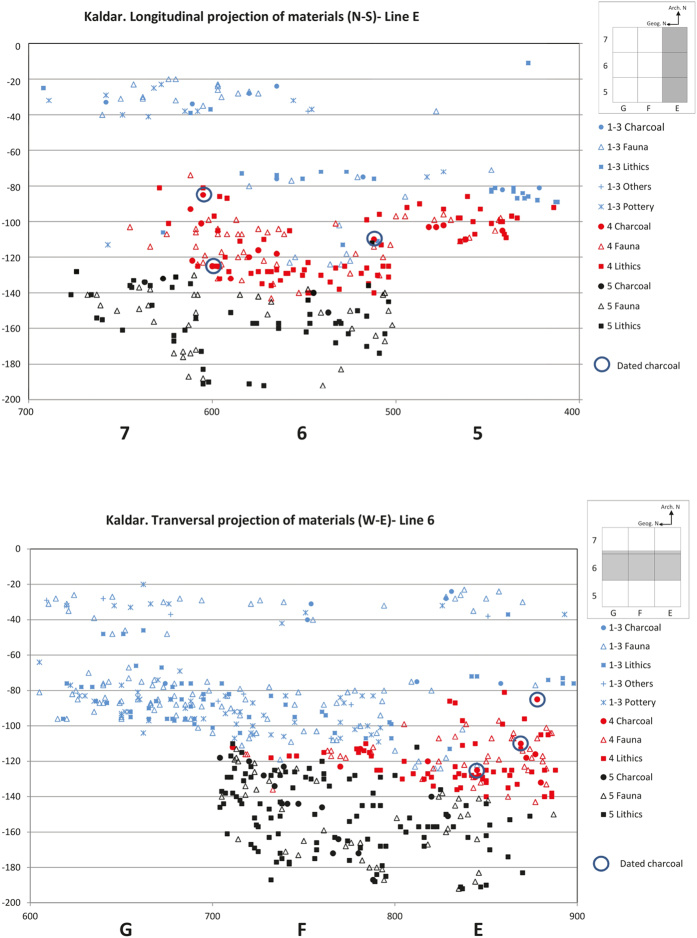
(**a**) North-south longitudinal projection of the materials from squares in line E. (**b**) West-east transversal projection of the materials from squares in line 6. Materials from Layers 1 to 3 have been projected together (blue). Projected separately are the materials from Layer 4 (red) and Layer 5 (black). Created by A. Ollé and B. Bazgir.

**Figure 3:**
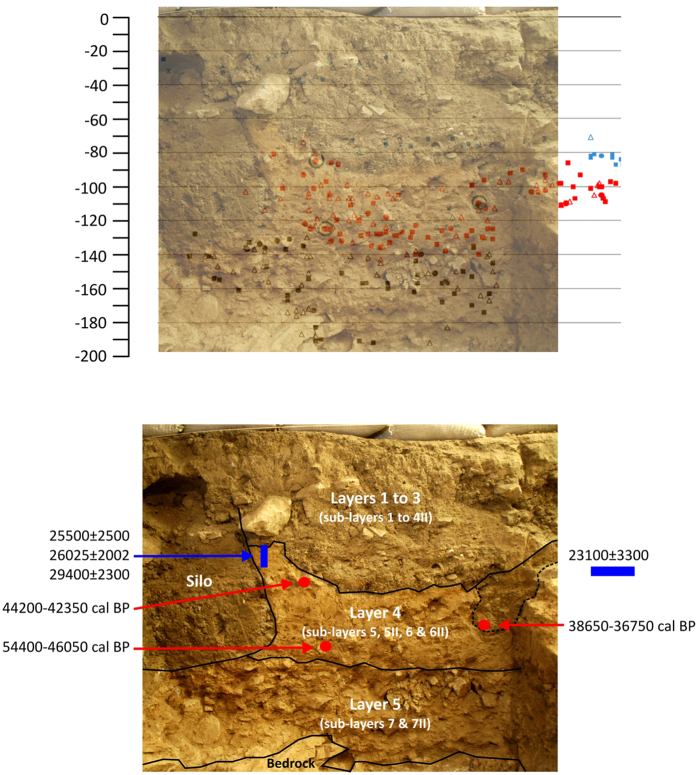
(Above) Stratigraphy (eastern section) along with transparent north-south longitudinal projection of the materials from squares in line E. (Below) Stratigraphy (eastern section) with location and results of the dated samples. Created by A. Ollé and B. Bazgir.

Layer 5 (including sub-layers 7 and 7II) consists of an extremely cemented reddish-brown sediment with some small angular limestone blocks and Middle Palaeolithic artefacts (Figs 2a,b and 3). To date, no radiometric data are available for this layer.

Bioturbation or disturbance was plotted, and sediment associated with the disturbance was removed without coordinating the finds, which were recorded as general finds with their approximate depths. Isolated evidence for intrusion below the Holocene layers was identified in a deep pit in square E7 in the upper part of the junction of sub-layers 5 and 5II. In the remainder of the site’s sequence, these layers are extremely hard and contain no evidence of bioturbation or disturbance. Heavy hammers and chisels were necessary to excavate these deposits (Supplementary Fig. S2). Consequently, we reached bedrock only in squares E6, E7, F6 and F7 (Supplementary Fig. S3).

### Faunal and floral remains

Bioarchaeological remains recovered to date allow us to make some initial environmental inferences and to correlate the faunal and the lithic records to reconstruct human subsistence activities.

A small portion of the faunal assemblage from Kaldar Cave was previously described^35^, but the recent excavations have yielded new material—some of which is described in the supplemental information (Supplementary Information, Supplementary Fig. S11). The preliminary study of the small vertebrates from Kaldar Cave has identified 218 remains coming from Layer 4 (sub-layer 5II) and Layer 5 (sub-layer 7II), comprising rodents, squamate reptiles, and amphibians. The updated faunal list is given in Table 3. There is no indication of a faunal change coincident with the cultural change from Layer 5 to Layer 4.

Most of the amphibians and reptiles (Agamidae, *Eryx* and Elapidae) live in savannah, steppe and desert environments and feature lifestyles linked to warm arid areas in rocky or sandy environments. *Pseudopus* lives in dry and bushy environments, sometimes in open woodlands, but avoids dense forest areas. The two most abundant rodent species in both Layers 4 and 5 are *Microtus* gr. *socialis* and *Meriones* spp., indicating that the environment surrounding the cave was composed mainly of dry open areas, with some vegetation cover, as indicated by the presence of Gliridae and Murinae taxa in both layers. *Cervus elaphus, Sus scrofa* and *Capreolus* may have preferred the more closed and humid environments in the valley near the river, whereas *Equus* may have favoured more open environments somewhat farther away on the flood plain (which could not have been very wide). Additionally, *Capra* may have lived in the higher areas. The region surrounding the cave was likely relatively humid close to the river and drier farther away, i.e., more or less similar to the modern conditions.

The charcoal assemblage shows the presence of *Prunus* (Layers 4 and 5) and *Salix* (Layer 5). This would suggest the presence of tree cover composed of willows near the river and open woodland possibly composed of several species including plum trees farther away. The presence of these taxa support the interpretation of an open woodland under mild climatic conditions inferred from the other proxies.

The animal species present in Kaldar Cave originated long before the Late Pleistocene. Some of the species show changes during this period, but the material from Kaldar Cave is not yet sufficient to assess the evolutionary level of these species. Thus, from this perspective, the fauna has limited biochronological value at the scale needed here.

A preliminary taphonomic analysis of the small mammal assemblage has shown a high number of digested elements, suggesting predation activity. According to the different degrees of digestion observed in the remains (light, moderate and some heavy), a category 3 predator, such as the tawny owl (*Strix aluco*) or the Eurasian eagle owl (*Bubo bubo*^36^), might be responsible. Both species are compatible with the inferred habitat and are present in the area today^37^. Additionally, both have opportunistic hunting habits and are sedentary; therefore, their prey well represents the local ecosystem.

The large vertebrates in Layers 4 and 5 are represented by highly fractured bones and teeth. Only seven complete remains (8.2% in Layer 4 and 7.1% in Layer 5) were recovered (1 unciform of *Capra*, 1 coracoid of *Testudo*, 1 tarso-metatarso of Aves from Layer 4; 2 teeth, 1 sesamoid of *Capra* and 1 caudal vertebra of a small mammal from Layer 5). Remarkably, approximately half of both sets (44.7% and 50%, respectively) are shaft fragments. An analysis of the fracture edges (according to Villa and Mahieu^38^) shows that breakage of the bones occurred when they were fresh because most fracture delineations are curved or v-shaped (59.4%) and longitudinal (36.8%), with oblique angles (85.8%). Despite the high degree of fracturing of the large vertebrate bones and teeth in Layers 4 and 5, the assemblages on the whole appear to be well preserved. Post-depositional modifications were generally scarce in the Kaldar assemblage, except for black stains from manganese oxide deposits, which were found on 24.1% of the Layer 4 remains and on 30.9% of the Layer 5 remains, and the cemented sediment attached to surfaces, which were found on 18.2% of the Layer 5 remains. These modifications suggest alternating damp and dry periods in the cave during the formation of Layer 5. Furthermore, sub-aerial weathering (stage 1 according to Behrensmeyer^39^) has been identified in just one specimen in each of the layers.

Evidence of anthropogenic activity appears in three ways: cut marks, bone fracturing, and cremations (Supplementary Fig. S4). Cut marks were observed on thirteen specimens: five from Layer 4 and eight from Layer 5. The remains from Layer 4 are long bones (humerus of Caprini, two tibia fragments and one femur of Cervidae and a long bone shaft of an indeterminate mammal). The cut marks appear in the form of slicing and scraping marks, and all instances are located on the shaft portions, indicating the defleshing of the carcasses. In Layer 5, the elements with cut marks comprise one radius and one tibia of *Capra*, one rib and one caudal vertebra of indeterminate taxa and four indeterminate long bone fragments. The incisions on the rib fragment were located on the neck of the bone and were associated with disarticulation activities. The incisions on the caudal vertebrae were located in the central part of the bone. The positions of the marks suggest that they are related to skinning tasks. Other bones have cut marks in midshaft positions, indicating defleshing of the carcasses. The *Capra* radius with cut marks also had an impact point produced by the anthropogenic breakage of the bone.

Among the anthropogenic modifications of the bones in Kaldar Cave, the most important are the changes in coloration due to cremation, which is present in all layers. Fully 23.2% of the remains of the assemblage are burned (24.1% in Layer 4 and 21.8% in Layer 5). These remains include charred (black coloured, 34.4%) and rubefacted (brown and red coloured, 9.4%) bones. Bones with multiple colours are also common (53.2% of the burned bones). The most common combination is rubefacted (brown) and charred (black) colours (46.9%) on the same bone, although partially calcined (grey-blue-white colours) specimens are also present. The presence of multiple colours on the surface of the bones has been associated with meat cooking^40^. The distribution of the colours is homogeneous on the surface and affects the fracture edge and the cortical and medullar faces, suggesting that the bones were burned after they had been broken. According to several experimental studies^40^,^41^,^42^, the presence of multiple colours indicates that the bones (regardless of the size) experienced cremation damage when they were fresh and unburied. The origin of this modification may be related to cooking but may also be related to their use as fuel for the maintenance of fires, cleaning of the living floor, or accidentally building a fire near the location where the bones had been deposited.

Little carnivore activity is recorded by the assemblage. Three bones in Layer 4 (1 tibia of Cervidae and 2 indeterminate long bones) showed carnivore tooth marks (Supplementary Fig. S4e). It is difficult to determine the size of the carnivore because only a few tooth marks are recorded. However, the low frequency of these modifications suggests carnivores played a limited role in the formation and/or modification of assemblage.

The zooarchaeological results suggest that not only were the early AMHs that occupied Kaldar Cave among the first to come into contact with large Palaearctic mammals but that they also quickly adapted to exploiting them as a resource.

### Lithic industry

The technological analysis of the archaeological samples associated with the Mousterian assemblage from Kaldar Cave (Layer 5 - sub-layers 7 and 7II) indicates that by-products (fragments and flake fragments) are the most common elements (12%) followed by retouched tools (10.8%), Levallois flakes (8.5%), cortical pieces (5.8%), Levallois blades (4%), Levallois points (2.4%), Levallois cores (0.8), other types of cores (0.8%) and hammerstones (0.4%). A large amount of debris (54.5%) is also present in the assemblage. The flakes are dominated by Levallois and cortical pieces, mostly with elongated morphologies and predetermined pointed shapes. Among the 82 flakes counted, 24.4% are cortical pieces, 24.4% are retouched, 20.7% have pointed shapes, 15.8% are broken, and 15.8% show enough major characteristics to be defined as a Levallois flake. Among the blade group, 37.1% are pointed in shape, 25.9% do not fit within a standard category, 14.8% are cortical, 11.1% are broken, 7.4% are retouched, and just one core (3.7%) was found. The retouched artefacts are dominated by marginal and broken retouched flakes (37%), Mousterian points (26%), different types of scrapers (24.1%), retouched points (5.6%), retouched blades (3.7%), Tayac points (1.8%), and limace (1.8%). The points (including Mousterian, Levallois, retouched and Tayac), along with pointed flakes and blades, comprise 11.4% of the entire assemblage in this layer. Among the material other than debris, the points and pointed elements comprise 25.1% of the assemblage in this layer. Mousterian points, Levallois points and retouched points comprise 2.8%, 2.4% and 0.6% of the assemblage, respectively. Not counting the debris, the Mousterian points and Levallois points comprise 6.2% and 5.3% the assemblage, respectively (Table 4).

**Figure 4:**
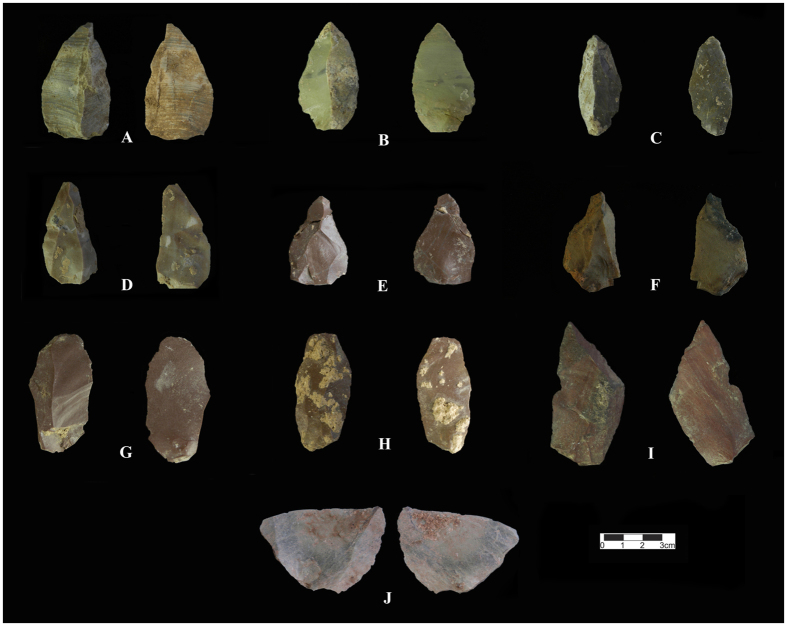
Selection of Middle Palaeolithic Levallois pieces from Kaldar Cave (Layer 5). (**A)** and (**B**); Point, (**C**); Elongated cortical point/Pointed flake with cortical butt, (**D**) to (**F**); Levallois point, (**G**) and (**H**); Elongated Levallois flake, (**I**); Levallois elongated pointed flake, (**J**); Levallois flake. Created by B. Bazgir.

The low number of cores (all exhausted) among the Mousterian assemblage from Kaldar Cave could be meaningful. This observation is in agreement with the techno-typological results from the Mousterian assemblage of the nearby Kunji Cave^43^. Given the notable scarcity of cores, the absence of refittable pieces, the large differences between the size of the tools and the size of the cores and their negative scars, and the condition of the cores that are exhausted, the *chaîne opératoire* is incomplete. Therefore, many of the artefacts were likely carried in from elsewhere (Fig. 4 and Supplementary Figs SI, S5, S9A & B, S10A & B).

**Figure 5:**
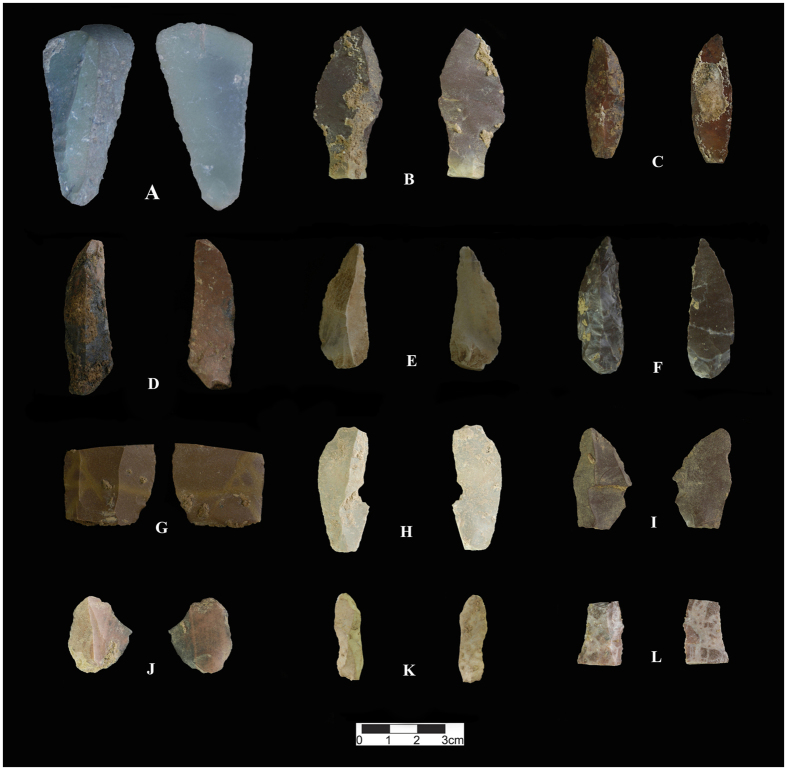
Selection of Upper Palaeolithic retouched pieces from Kaldar Cave (Layer 4). (**A**); Cortical retouched double scraper, (**B**); Tanged point, (**C)** to (**F**); Arjeneh points, (**G**); Retouched blade, (**H**); Elongated retouched blade, (**I**); Point on blade with retouches on its distal portion of ventral face, (**J**); Retouched end scraper, (**K**); Retouched nosed scraper, (**L**); Mesial portion of a retouched bladelet point. Created by B. Bazgir.

**Figure 6:**
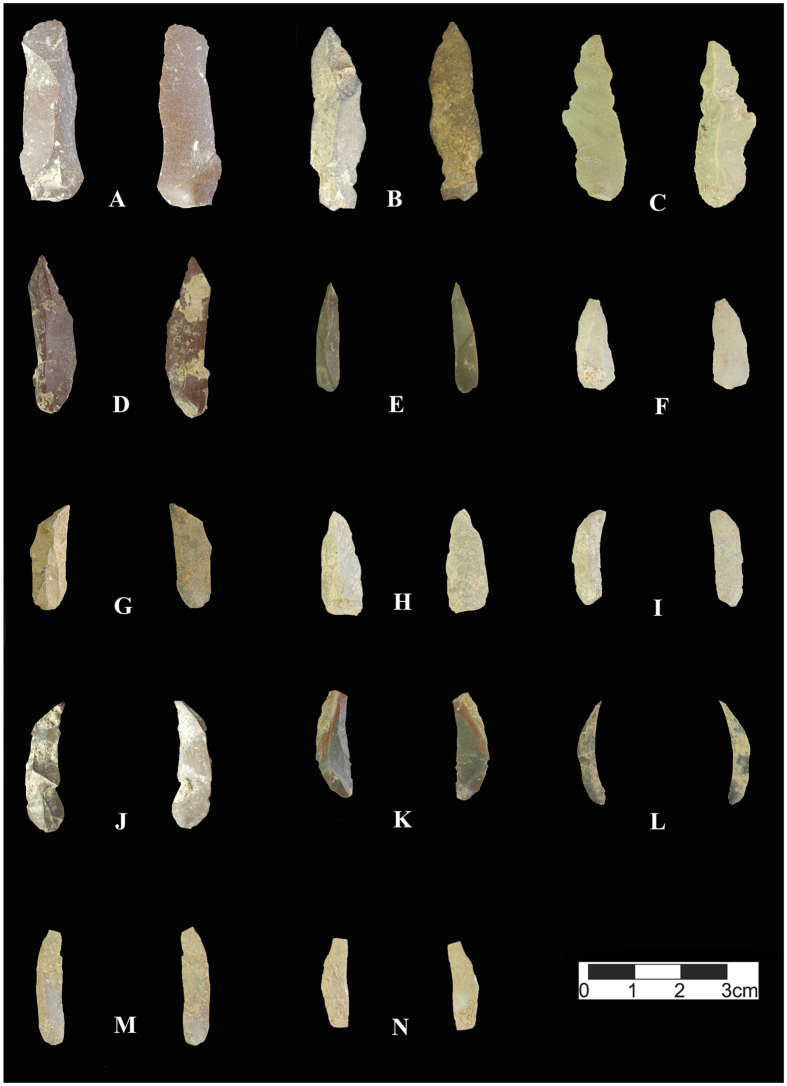
Selection of Upper Palaeolithic blades and bladelets from Kaldar Cave (Layer 4). (**A**) Elongated blade, (**B)** to (**D**); elongated pointed blades, (**E**) to (**H**); Pointed bladelets, (**I**) and (**N**); Dufour bladelets, (**J**) to (**M**); Twisted bladelets. Created by B. Bazgir.

**Figure 7:**
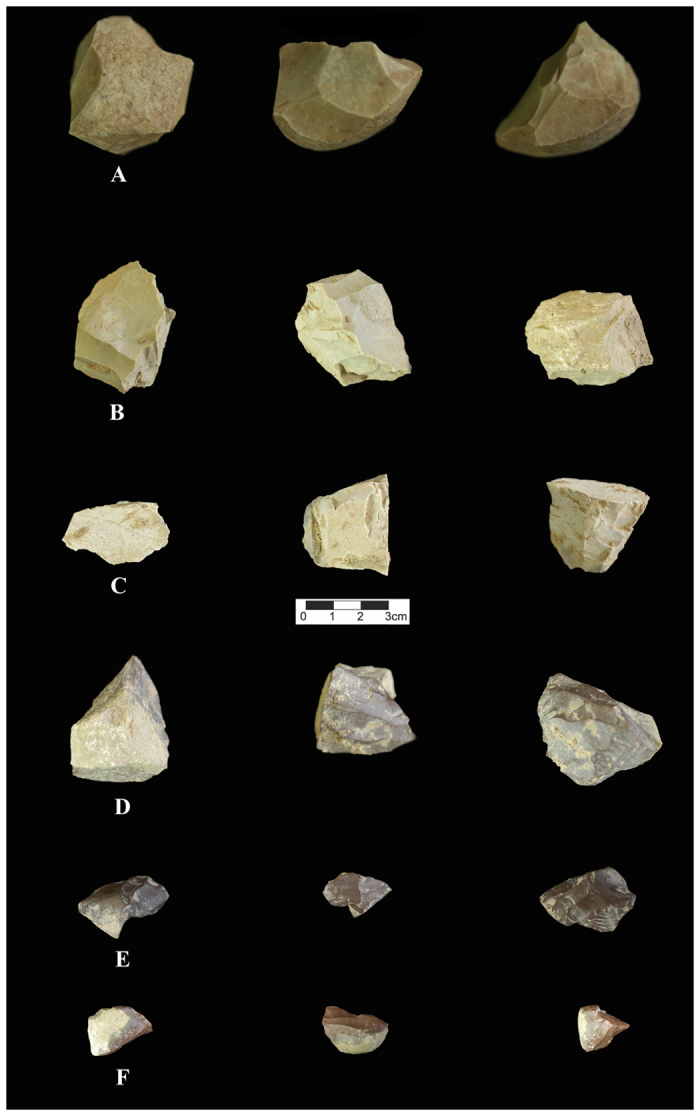
Selection of Upper Palaeolithic cores from Kaldar Cave (Layer 4). (**A**): Flake core, (**B**,**C** and **F**); Bladelet core, (**D**); Broken carinated core (**E**); Carinated core/carinated scraper. Created by B. Bazgir.

In the Upper Palaeolithic lithic assemblages of Layer 4 (sub-layers 5 & 5II), bladelets dominate (13%), followed by blades (12.5%), retouched tools (5.1%), cortical pieces (4.4%), by-products (3.5%), bladelet cores (1.6%), undetermined cores (1.4%; including a centripetal core), pointed flakes, blanks, and other types of tools (a borer and point; all less than 1%), a blade core (0.2%) and finally a considerable amount of debris (56.4%). Within the bladelet categories, there is a good representation of twisted bladelets (14.3%). Among the retouched tools, Arjeneh points are abundant, but pointed pieces (including Tanged, retouched points, pointed blades and bladelets and Arjeneh points) are more numerous (54.5%) compared to other types of retouched tools (Figs 5, 6 and 7, and Supplementary Figs S6, S7 and S8). Excluding the debris in this layer, the points and pointed elements comprise 11.2% of the entire assemblage. The next most abundant tools among the retouched pieces are the scrapers (including side-scraper, end scraper and nosed scraper), representing 18.2% of the tools. The number of flakes in this layer is very low (4.6% of the assemblage), and among the flakes, 3.7% are cortical flakes, 0.7% are pointed flakes and 0.2% are retouched flake (Table 5).

Despite the small size of the assemblage, a quick examination of the assemblage data from both Layer 5 (sub-layers 7 & 7II) and Layer 4 (sub-layers 5, 5II, 6 & 6II) shows a significant technological change from flake technology towards the production of blades and bladelets. However, to be more precise, a preliminary comparative analysis between the two layers was performed (Supplementary diagrams S1 to S5). In this analysis, we compared the weights and average values of metric measurements of various characteristics and attributes. The comparison of comparable categories was performed to provide meaningful results and to aid our interpretation of these two layers. Therefore, we compared Levallois cores vs. blade/bladelet cores, pointed blades vs. pointed blade/bladelets, and the retouched points, cortical pieces and cortical flakes (within the cortical pieces) from both the layers. Interestingly, our comparative analysis shows that significant differences are present among all the elements from the Middle and Upper Palaeolithic industries of Kaldar Cave. The weights and sizes of all the compared elements tend to be greater in Layer 5 than in Layer 4. The only exception was found within the retouched points. In this case, the average length and thickness are slightly greater for Layer 4 than for Layer 5.

## Discussion

Considerable efforts have been made to address fundamental questions concerning the cultural remains associated with AMHs. These attempts have been based mainly on Upper Palaeolithic lithic assemblages and their potential places of origin.

Based on technological comparisons between the lithic assemblages found in Europe and those found in the Zagros, some authors report close typological similarities between the two and further propose the latter region as the most probable source of the technology, with an east-to-west diffusion into Europe. Consequently, the Upper Palaeolithic tradition of the Zagros has been termed the ‘Zagros Aurignacian’^12^,^13^,^14^,^19^,^20^,^21^,^23^,^24^,^25^,^26^,^27^,^44^. Based on the techno-typological analysis of material from Warwasi, some also claim that the Baradostian (or Zagros Aurignacian) technology evolved from a local Mousterian foundation in the area.

In conflict with the statement by Tsanova^15^ that the Iranian Zagros cannot be the source of bladelet technology and cultural modernity as the Warwasi rockshelter lacks both radiocarbon dates and evidence of antecedent blade technology, strong evidence indicates that the Zagros assemblages are not merely blade-based. Over 90 sites contain evidence of clear blade(let) production (defined as the Rostamian tradition), and these tools are all similar to and associated with those from the well-stratified Ghare-Boof locality^8^,^9^,^10^,^11^.

Based on the detailed techno-typological analysis of the industries from Yafteh, some authors claim that the Baradostian technology of the Zagros is an Early Ahmarian-like technology^45^,^46^ and conclude that, on the basis of the available data, continuity from the Zagros Mousterian to the Zagros Aurignacian cannot be confirmed. However, based on the gradual transition from the Middle Palaeolithic to the Upper Palaeolithic at Warwasi and the technological and chronological analogies between the lower Baradostian at Yafteh and the Early Ahmarian, the Zagros region remains a potential candidate for the origin of the Aurignacian^5^,^32^. In a very recent typo-technological study on the Ghar-e-Khar lithic assemblages^33^, the authors estimated the potential of the area for future research. Nevertheless, in addition to the small sizes of the studied assemblages, methodological problems (e.g., using 10- to 30-cm arbitrary levels) during the excavation and the lack of absolute chronometric data might raise concerns similar to those for the material from Warwasi and cast doubt on the results from Ghar-e-Khar, which are not compelling. Thus, the hypothesis of Middle-to-Upper Palaeolithic continuity in Zagros and the possibility of a gradual transition are hard to assess due to the current state of the technological data^33^.

Similar to Yafteh, the Üçağizli sequence in Turkey provides some evidence of evolution from the Initial Upper Palaeolithic (IUP) into the early Upper Palaeolithic “Early Ahmarian”. Given the absence of Middle Palaeolithic underlying the IUP layers in Üçağizli, however, the site offers little to the discussion of the appearance of the IUP in the region^47^ (see also Shidrang^32^). Additionally, an IUP assemblage has also been discovered in Manot Cave, to the north of Mount Carmel^48^. The presence of both Mousterian and Baradostian cultural remains in Kaldar Cave and the recent chronometric data can be used to address many of the stated uncertainties associated with the transition process.

In regard to the terms “IUP”, “Aurignacian”, “Baradostian” and “Zagros Aurignacian”, our data from Kaldar Cave and other excavated localities^35^ support the arguments advanced by Kuhn and Zwyns^49^ with respect to the technological diversity within the assemblages and the long duration of the Upper Palaeolithic in Kaldar. We therefore avoid using the term “IUP” for this assemblage. On the other hand, we cannot simply assign the term “Aurignacian” to the assemblage based on certain similarities with assemblages from European sites. However, our observations and technological analysis of the Kaldar assemblage are in agreement with that of Olszewski^12^,^13^,^14^,^19^,^20^,^21^,^22^,^23^,^24^,^44^: certain similarities do exist, yet the Zagros industry differs from the purely European Aurignacian. Therefore, to us, the terms “Baradostian” or “Zagros Aurignacian” are more appropriate.

Notably, based on our earlier technological work^35^, the recent TL dates are older than we anticipated for the lithic assemblages of the uppermost part of Layer 4. These dates have led us to abandon the Epipalaeolithic designation we previously applied to these bladelet assemblages.

The AMHs in Kaldar Cave may have been among the first of their kind to interact with Palaearctic fauna. Thus, many of the species were new to them. In this part of Eurasia, the Palaearctic had an east-west-oriented southern border with the newly defined Saharan-Arabian biogeographic realm. The Zagros Mountains acted as an extension of the Palaearctic into the more southern realm^50^. However, it is not known whether the boundary between these realms occupied the same location during the Late Pleistocene. The presence of large mammals is indicative of the seasonality of the Palaearctic, but most of the reptiles have Saharan-Arabian affinities, and the rodents yield a mixed signal.

The fauna present in the mid-latitude Palearctic represent “interglacial” fauna, and similar faunas (albeit generally richer in species) occupied the area during previous interglacials. During glacial periods, these species survived in southern refugia, while cold-adapted species occupied the mid-latitudes. Iran may have acted as one of these refugia. Up to now, no typically glacial species has been recorded in Iran or other areas at similar latitudes. The Palaearctic mammal species recorded in Iran, and in particular in Kaldar Cave, are “interglacial”, suggesting the presence of at least temperate conditions. In contrast, the herpetofauna clearly indicates warm conditions. This combination is consistent with a position at the limit of the two biogeographic realms during climatic conditions similar to those of today. Furthermore, the study period is thought to correspond to MIS3, which had conditions similar to the modern climate. Because there is no indication of faunal change between layers 4 and 5, the available evidence suggests that the cultural change was not related to climatic or environmental changes.

## Methods

### Radiocarbon dating

Radiocarbon dating of the five charcoal samples (listed in Table 4) was performed at the Oxford Radiocarbon Accelerator Unit (ORAU). The samples were chemically cleaned using the acid-base-wet oxidation-stepped combustion (ABOx-SC) protocol (after Brock and Higham^51^, also see ref. 52) or a modification of the same. The ABOx-SC method was employed as it has been shown to remove contaminants from Palaeolithic-aged charcoal more efficiently than the routine acid-base-acid (ABA) protocol, often yielding significantly older dates (e.g. refs 51, 53, 54, 55, 56, 57, 58, 59). The analytical data obtained are shown in Table 4, and no data fall outside the expected ranges for well-preserved charcoal. The calibration of all the resulting AMS radiocarbon determinations was performed using the OxCal 4.2 software^60^,^61^ and the IntCal13 calibration curve^62^.

**Figure 8:**
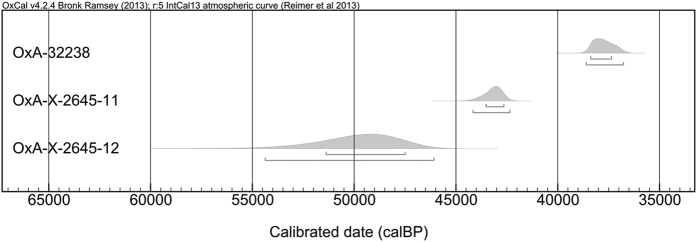
Calibrated results of the Palaeolithic specimens. Created and modified in the Research Laboratory for Archaeology and the History of Art, University of Oxford, by T. Higham, K. Douka and L. Becerra-Valdivia.

Among the seven charcoal samples submitted, five yielded enough material for AMS radiocarbon dating after chemical preparation (see Table 6). Only three of these, however, yielded reliable radiocarbon dates following a congruent age-depth pattern; the two others were substantially younger. This is almost certainly due to taphonomic influences. While it would be useful to incorporate the Palaeolithic-aged results into a Bayesian model, we cannot as we have too few results at this time. More dating work is currently underway, and we hope to be able to report new results in the future. In Fig. 8, we show the calibrated results for the Palaeolithic specimens (see Table 2 for the data).

### TL dating

Thermoluminescence dating was performed on five heated samples (four heated sediments from two fire places in the upper most part of Layer 4 and one burnt flint from Layer 5) at the Research Centre for Conservation & Restoration of Cultural Relics of the Research Institute of Iranian Cultural Heritage (RICHT). At present, the samples from Layer 4 have successfully been dated. Three of the dated samples come from a fireplace within squares E6/7 and one from square E5 (Table 1).

For the sample preparation and instrumentation, the outer surface (3 mm) of the samples was removed. To account for the alpha radiation contribution to the natural dose measurements, the fine grain technique is used (ibid). Alpha radiation travels an extremely short distance in heated objects (approximately 25 μm^63^). Thus, we used grains less than 10 μm in size. The samples were crushed and treated with 10% HCl to remove carbonates and organic material. Then, all samples were washed with distilled water and then with acetone. Finally, the grains were suspended in acetone and deposited on aluminium discs that were 10 mm in diameter and 0.5 mm in thickness.

The TL measurements were performed using an ELSEC7188 instrument. The potassium contents of the samples were determined by flame photometry. To determine the contributions from U and Th, the “pairs” technique was used; thus, the dose rate was measured using a 7286 low-level alpha counter^64^. External dose rates were measured by in situ dosimetry^65^. The CaF2 TL-Dosimeter was located in site for 36 days. These values were calculated for different levels, up-level: 0.787 mGy/a, down-level: 0.660 mGy/a. Measurements of the water content and fading test for all samples were considered (Table 1).

## Conclusions

The newly excavated sequence in Kaldar Cave provides evidence for the replacement of the Mousterian industry, usually associated with Neanderthals, by the Baradostian industry, similar to the Aurignacian, which is unique to anatomically modern humans. Radiocarbon dates suggest that this may have occurred prior to 49,200 ± 1800 BP, probably during the relatively warm MIS3. The faunal evidence is consistent with the replacement occurring during MIS3 and does not support a coincident climatic change. Kaldar Cave is situated in the southernmost part of the Palaearctic biogeographic realm. Evidence from Kaldar Cave is among the oldest to show that AMHs were capable of exploiting the Palaearctic fauna and were thus well adapted to this new environment, which they colonized shortly after the period of time recorded in the cave.

Excavations at Kaldar Cave have yielded evidence for Baradostian (Layer 4) and Mousterian assemblages (Layer 5) in stratigraphic superposition. This is an exceptional find in the Zagros. The preliminary technological analysis on the lithic industry from both layers indicates a clear shift from flake production to blade and bladelet technology. Furthermore, despite the small size of the lithic assemblage so far, the quantitative comparative analysis shows a significant difference between elements within the Middle and Upper Palaeolithic layers. The homogeneity of the differences between all the compared elements—that is to say, the greater weight and size of the items in the Mousterian assemblage compared to those of the Upper Palaeolithic assemblage—could be a reliable foundation for interpretation and understanding the two industries.

We have obtained new chronometric data from the site. Four TL dates from the uppermost Layer 4 revealed ages that ranged from 23100 ± 3300 to 29400 ± 2300 BP.

The three ^14^C dates from Layer 4 and sub-layers 5 and 5II produced results in the ranges of 38650–36750 cal BP, 44200–42350 cal BP, and 54400–46050 cal BP, respectively (all at 95.4% probability). The wide chronometric ranges and few dates do not allow us to make a confident and precise assessment of the age of the transition to the Upper Palaeolithic. Further work is needed to refine the chronology.

In addition to the presence of a clear Mousterian industry in the >0.5-m-thick Layer 5 and despite the need for more chronometric data, the obtained dates from the lower part of the Upper Palaeolithic sequence in Kaldar Cave are some of the earlier dates attributed to a lithic industry produced by AMHs in western Asia. Although we do not intend to challenge the Levantine dispersal theory, previous work has noted that the Aurignacian may not have originated in only one area^22^ (see also Groucutt^66^). It has been suggested that the ages of the so-called “transitional” or Initial Upper Palaeolithic layers at Ksar Akil may represent that the transition from the Middle to Upper Palaeolithic in this area (and possibly in the wider northern Levant) occurred later than previously estimated. This finding would cast doubt on the assumed singular role of the region as an origin for human dispersal into Europe^67^.

Another important clue derived from the preliminary quantified results of the Mousterian and Upper Palaeolithic lithic industries in Kaldar Cave is the high percentage of points and pointed elements in both the layers. This abundance may indicate that the site functioned as an important hunting camp in the Zagros Mountains during both the Middle and Upper Palaeolithic times. This hypothesis appears to be supported by the zooarchaeological evidence. Hence, Kaldar Cave provides one of the oldest examples of modern human existence in this part of the world and provides data on how these populations coped with the Palearctic climatic and environmental situations, which were new to them.

To reach a consensus regarding the Middle to Upper Palaeolithic transition/continuity, several lines of evidence are required. Indeed, accurate information and maximum control of the context, including careful sampling for chronometric dating from well-stratified sites and detailed techno-typological analysis, are crucial factors. Our understanding of the behavioural dimension of the transitional phenomenon would also benefit from more excavations using multidisciplinary methods, including spatial analysis and functional aspects.

## Additional Information

**How to cite this article:** Bazgir, B. *et al*. Understanding the emergence of modern humans and the disappearance of Neanderthals: Insights from Kaldar Cave (Khorramabad Valley, Western Iran). *Sci. Rep.* **7**, 43460; doi: 10.1038/srep43460 (2017).

**Publisher's note:** Springer Nature remains neutral with regard to jurisdictional claims in published maps and institutional affiliations.

## Supplementary Material

Supplementary Information

## Figures and Tables

**Figure 1:**
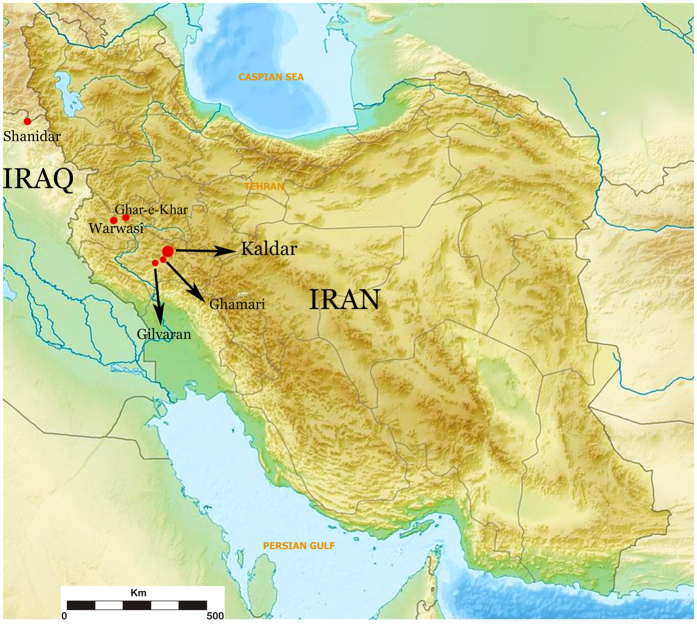
The excavated sites containing Middle Palaeolithic and early Upper Palaeolithic sequences in the Zagros. (Source of the original map: https://commons.wikimedia.org/wiki/File:Iran_relief_location_map.jpg (under the license of Creative Commons Attribution-Share Alike 3.0 Unporte). Modified by B. Bazgir. Original license pages: https://en.wikiedia.org/wiki/Creative Commons - https://creativecommons.org/licenses/by-sa/3.0/deed.en.

**Table 1:** List of thermoluminescence dating results from Kaldar Cave.

Sample	Specifications	Th	Ur	K2%	Equivalent Dose (ED)	Age
1	Layer 4: sub-layer 5, E6-7	1.76	3.95	0.81	73.64	26025 ± 2002
2	Layer 4: sub-layer 5, E6-7	2.94	1.86	0.97	51.97	29400 ± 2300
5	Layer 4: sub-layer 5, E6-7	2.46	5.54	1.29	178.79	25500 ± 2500
4	Layer 4: sub-layer 5, E5	1.51	3.30	1.19	64.85	23100 ± 3300

**Table 2:** Radiocarbon results for charcoal samples from Kaldar Cave.

Sample	OxA-	Archaeological context	δ^13^C (‰)	Conventionl radiocarbon age (BP)	Calibrated date (95.4% probability)
723	32238	Trench (T) 1; Layer 4, sub-layer 5; SQ E6; 69 (X), 12 (Y), 110 (Z)	−23.0	33,480 ± 320	38650–36750 cal BP
—	32239	T1; Layer 4, sub-layer 5; SQ G6	−23.1	964 ± 26	1000–1200 AD
—	32240	T1; Layer 5, sub-layer 7II; SQ F7	−27.1	1.09665 ± 0.00323**	1850–1950 AD
274	X-2645-11	T 1; Layer 4, sub- layer 5; SQ E7; 78 (X), 5 (Y), 85 (Z)	−23.4	39,300 ± 550	44200–42350 cal BP
869	X-2645-12	T1; Layer 4, sub-layer 5II; SQ E6; 45 (X), 100 (Y), 125 (Z)	−24.5	49,200 ± 1800	54400–46050 cal BP

**Table 3:** Distribution of the faunal taxa identified in Kaldar Cave, Layers 4 and 5.

	Layer 4	Layer 5
Mammals		
**Carnivora**		
Mustelidae indet.		x
**Perissodactyla**		
*Equus* sp. (horse)	x	
**Artiodactyla**		
*Sus scrofa* (wild boar)	x	
*Capreolus* sp. (roe deer)		x
*Cervus elaphus* (red deer)	x	x
*Capra* cf. a*egagrus* (goat)	x	x
**Rodents**		
*Microtus* gr. *socialis* (social vole)	x	x
*Chionomys* cf. *nivalis* (European snow vole)	x	
*Ellobius* cf. *lutescens* (Transcaucasican mole vole)		x
*Ellobius* cf. *talpinus* (northern mole vole)	x	
*Ellobius* sp. (mole vole)	x	x
*Cricetulus* cf. *migratorius* (migratory hamster)	x	x
*Mesocricetus* cf. *brandti* (Turkish hamster)	x	x
*Calomyscus* sp. (mouse-like hamster)		x
*Meriones* spp. (two morphotypes of gerbil)	x	x
Cf. *Allactaga* sp. (toad jeroba)		x
*Myominus* sp. (mouse-tailed dormouse)		x
*Dryomys* cf. *nitedula* (forest dormouse)		x
*Apodemus* cf. *flavicollis* (yellow-necked mouse)	x	x
*Mus* cf. *musculus* (house mouse)	x	x
**Birds**		
Aves indet.	x	
**Reptiles**		
Agamidae indet. (agamid lizard)	x	x
Gekkonidae indet. (gecko)		x
Scincidae indet. (skink)		x
Lacertidae indet. (lacertid lizard)	x	x
*Pseudopus* sp. (glass lizard)		x
*Eryx* sp. (sand boa)	x	x
Colubrinae indet. (6 morphotypes)		x
Elapidae indet. (cobra)		x
Viperidae indet. (viper)	x	x
*Testudo* sp. (tortoise)	x	
**Amphibians**		
*Bufo* sp. (toad)		x
Anura indet.	x	
**Crustaceans**		
Crustacea indet. (crab)	x	

**Table 4:** Quantified results of the lithics attributed to the Middle Palaeolithic Layer 5 of the 2014–2015 excavation season at Kaldar Cave.

Layer 5 (sub-layers 7 and 7II)			N	%
**Cortical piece**	Cortical flake	20	29	**5.8**
	Cortical elongated point	1		
	Cortical blade	4		
	Pebble	1		
	Cortical scraper	3		
**Levallois flake**	Levallois flake	13	42	**8.5**
	Retouched flake	(20 counted in retouched tools)		
	Pointed flake	16		
	Broken flake	13		
**Levallois blade**	Pointed blade	10	20	**4**
	Cortical blade	(4 counted in cortical pieces)		
	Retouched blade	(2 counted in retouched tools)		
	Broken blade	3		
	Others	7		
**Levallois point**	—	12	12	**2.4**
**Levallois core**	—	4	4	**0.8**
**Other types of core**	Undetermined core	2	4	**0.8**
	Discoid core	1		
	Blade core	1		
**Retouched tool**	Mousterian point	14	54	**10.8**
	Marginal and broken retouched flake	20		
	Retouched point	3		
	Scraper	7		
	Nosed scraper	1		
	Side scraper	1		
	Retouched blade	2		
	Cortical scraper	4		
	Limace	1		
	Tayac point	1		
**Byproduct**	—	60	60	**12**
**Debris**	—	271	271	**54.5**
**Hammerstone**	—	2	2	**0.4**
**Total**	—	498	498	**100%**

**Table 5:** Quantified results of the lithics attributed to the Upper Palaeolithic Layer 4 of the 2014–2015 excavation season at Kaldar Cave.

Layer 4 (sub-layers 5, 5II, 6 and 6II)			N	%
**Cortical piece**	Cortical flake	16	19	**4.4**
	Cortical blade	1		
	Nodule	2		
**Blade**	Pointed blade	6	54	**12.5**
	Blade with truncated faceted butt	1		
	Blade	47 (2 counted in retouched tool)		
**Bladelet**	Twisted bladelet	8	56	**13**
	Bladelet point	2		
	Bladelet	46 (5 counted in retouched tools)		
**Blade core**		1	1	**0.2**
**Bladelet core**		7	7	**1.6**
**Other types of core**		6 (1 is a centripetal core)	6	**1.4**
**Retouched tool**	Nosed scraper	1	22	**5.1**
	End scraper on blade	1		
	Blade scraper	1		
	Side scraper	1		
	Arjeneh point	4		
	Tanged point	1		
	Retouched pointed bladelet	3		
	Retouched bladelet	2		
	Retouched pointed blade	2		
	Retouched point	2		
	Unfinished retouched point	1		
	Retouched flake	1		
	Retouched blade	1		
	Retouched piece on a broken blade	1		
**Other types of tool**	Borer	1	1	**0.3**
**Pointed flake**		4	4	**0.9**
**Blank/fragment**		3	3	**0.7**
**Byproduct**		15	15	**3.5**
**Debris**		243	243	**56.4**
**Total**			431	**100%**

**Table 6:** Analytical and sample data for the charcoal dated from Kaldar Cave.

OxA	Species	Used (mg)	Yield (mg)	%Yld	%C	δ^13^C (‰)
X-2645-11	*Prunus* cf. *amygdalus*	59.91	8.71	14.5	81	−23.4
X-2645-12	*Prunus* cf. *amygdalus*	93.41	6.23	6.7	82.2	−24.5
32238	*Prunus* cf. *amygdalus*	107.6	10.81	10	69.7	−23.0
32239	*Quercus* sp. deciduous	105.16	16.65	15.8	71.7	−23.1
32240	*Quercus* sp. deciduous	9.83	6.5	66.1	75.1	−27.1

OxA-X determinations involved a modified ABOx-SC preparation. The other determinations involved the ABOx-SC method. All data are acceptable and within expected parameters. **Denotes an AMS result in fraction Modern. This determination post-dates AD 1950.
