# Supplementary material for: Understanding the emergence of modern humans and the disappearance of Neanderthals: Insights from Kaldar Cave (Khorramabad Valley, Western Iran)
Source: Sci Rep. 2017 Mar 2;7:43460. doi: 10.1038/srep43460 (PMC5333163; doi:10.1038/srep43460)
Supplement: Supplementary Information [file srep43460-s1.pdf]

SUPPLEMENTARY INFORMATION

Understanding the emergence of modern humans and the disappearance of Neanderthals: Insights from Kaldar Cave (Khorramabad Valley, Western Iran)

Behrouz Bazgir[1,2], Andreu Ollé[1,2], Laxmi Tumung[2,1], Lorena Becerra-Valdivia[3], Katerina Douka[3], Thomas Higham[3], Jan van der Made[4], Andrea Picin[5,6,1], Palmira Saladié[1,2,7,8], Juan Manuel López-García[1], Hugues-Alexandre Blain[1,2], Ethel Allué[1,2,7], Mónica Fernández-García[9], Iván Rey-Rodríguez[1], Diego Arceredillo[10], Faranak Bahrololoumi[11], Moloudsadat Azimi[11], Marcel Otte[12], Eudald Carbonell[2,1].

[1]Institut Català de Paleoecologia Humana i Evolució Social (IPHES), Zona educacional 4, Campus Sescelades URV (Edif. W3), 43007 Tarragona, Spain.
[2]Àrea de Prehistòria, Universitat Rovira i Virgili. Fac. de Lletres, Avinguda Catalunya 35, 43002 Tarragona, Spain.
[3]Oxford Radiocarbon Accelerator Unit, Research Laboratory for Archaeology and the History of Art, University of Oxford, Dyson Perrins Building, South Parks Road,OX1 3QY Oxford, United Kingdom.
[4]CSIC, Museo Nacional de Ciencias Naturales, c. José Gutiérrez Abascal 2, 28006 Madrid, Spain.
[5]Bereich für Ur- und Frühgeschichtliche Archäologie, Friedrich Schiller Universität Jena, Löbdergraben 24a, Jena, 07743 Germany.
[6]Neanderthal Museum, Talstrasse 300, D40822, Mettmann, Germany.
[7]GQP-CG, Grupo Quaternario e Pre Historia do Centro de Geociencias (uI&D 73 e FCT), Portugal.
[8]Unit Associated to the Centro Superior de Investigaciones Científicas (CSIC), 28006, Madrid, Spain.
[9]Sezione di Scienze Preistoriche e Antropologiche, Dipartimento di Studi Umanistici, Università degli Studi di Ferrara (UNIFE), C. so Ercole I d'Este 32, 44121 Ferrara, Italy.
[10]Facultad de Humanidades y Ciencias Sociales, Universidad Internacional Isabel I de Castilla, c. Fernán González 76, 09003 Burgos, Spain
[11]Iran's Research Institute for Cultural Heritage and Tourism, Emam's square, 11369 -13431, Tehran, Iran.
[12]University of Liège, Service of Prehistory, place du 20-Août 7, A1, 4000 Liège, Belgium.

Description of the new material of large mammals from Kaldar Cave

Remains of large mammals have been described from a previous excavation at Kaldar Cave[1]. Recent excavations yielded new material, including new species. The updated faunal list is given in Table 3. The most relevant new fossils are described below.

An incisor (Fig. S11/2) from Layer 4 (sub-layer 5 II) belongs to *Equus*. Its size suggests a large horse, such as the caballoid horses, and not a small one such as *E. hydruntinus* or *H. hemionus*. Caballoid horses are seen as a single species[2], two[3] or even more species[4]. We assign the material to *Equus* sp.

A right upper canine of a cervid comes from Layer 5 (Fig. S11/1). The genera *Axis* and *Rucervus* do not have upper canines, the genus *Dama* only rarely has them[5], but in *Cervus elaphus*, both males and females have upper canines. By the criteria of D'Errico & Vanhaeren[6] and Arceredillo[7], the specimen from Kaldar belonged to a female, but it is not possible to infer the age of death. In Europe, the size of *Cervus elaphus* changed in time and the same changes seem to have occurred even South of the Caucasus[8,9,10]. However, the red deer material from Kaldar Cave does not allow to establish whether it belonged to a large or small sized population.

Caprini indet. cf. *Capra aegagrus* was previously identified on the basis of dental material. A lower third molar from Layer 5 (sub-layer 7) (Fig. S11/3) is peculiar in having an additional distal lobe. Normally there is a third lobe, consisting of a single cusp, but here there is a relatively wide third lobe, consisting of a single cusp, and a similar but narrower fourth lobe. The fourth lobe is worn by occlusion with the upper M^3. *Hemitragus* and *Capra* differ in that the latter has upper third molars with a distal

extension at the buccal side, which is like a very narrow third lobe. *Hemitragus* and nearly all other bovids lack such an incipient third lobe. A short M^3, without such an extension, would never cause wear on the tip of a fourth lobe of an M_3. This tooth confirms thus the presence of *Capra* in Kaldar Cave.

References

1: Bazgir, B. *et al*. Test excavations and initial results at the Middle and Upper Paleolithic sites of Gilvaran, Kaldar, Ghamari caves and Gar Arjene Rockshelter, Khorramabad Valley, western Iran. *CR Palevol*. **13**, 511–525 (2014).

2: Azzaroli, A. The genus *Equus* in Europe. In *European Neogene Mammal Chronology* (ed. Lindsay, E.H., Fahlbusch, V. and Mein, P) 339-356 (Plenum Press, 1990).

3 : Forstén, A. Middle Pleistocene replacement of stenonid horses by caballoid horses - ecological implications. *Palaeogeogr. Palaeoclimatol. Palaeoecol*. **65**, 23-33. (1988).

4 : Eisenmann, V. Les Chevaux Quaternaires européens (Mammalia, Perissodactyla). Taille, typologie, biostratgraphie et taxonomie. *Geobios*, **24**, 747-759. 1991

5: Groves, C. and Grubb, P. *Ungulate Taxonomy*. (The Johns Hopkins University Press, 2011).

6 : d'Errico, F. and Vanhaeren, M. Criteria for identifying red deer (*Cervus elaphus*) age and sex from their canines. Application to the study of Upper Palaeolithic and Mesolithic ornaments. *J. Arch. Sci*. **29**, 211-232 (2002).

7: Arceredillo, D. *Estudio Paleobiológico de los ungulados del Pleistoceno superior de la Meseta Norte*. Unpublished PhD thesis, Universidad de Salamanca (2015).

8: Made, J. van der. Observations on the fauna from Terra Amata. In *Terra Amata, Nice, Alpes-Maritimes, France. Tome II. Palynologie, Anthracologie, Faunes des vertébrés, Molusques, Paléoenvironnements, Paléoanthroplogie* (ed. De Lumley, H.) 277-284 (CNRS Éditions, 2011).

9 : Made, J. van der, Stefaniak, K. and Marciszak, A. The evolution and Polish fossil record of *Canis*, *Alces*, *Capreolus*, *Megaloceros*, *Dama* and *Cervus*. *Quat. Intern*. **326-327**, 406-430 (2014).

10: Made, J. van der *et al.* The new material of large mammals from Azokh and comments on the older collections. In *Azokh caves and the transcaucasian corridor*: (ed. Fernández-Jalvo, Y., King, T., Andrews, P. and Yepiskoposyan, L.) 117-162 (Springer, 2016).

Supplementary figures

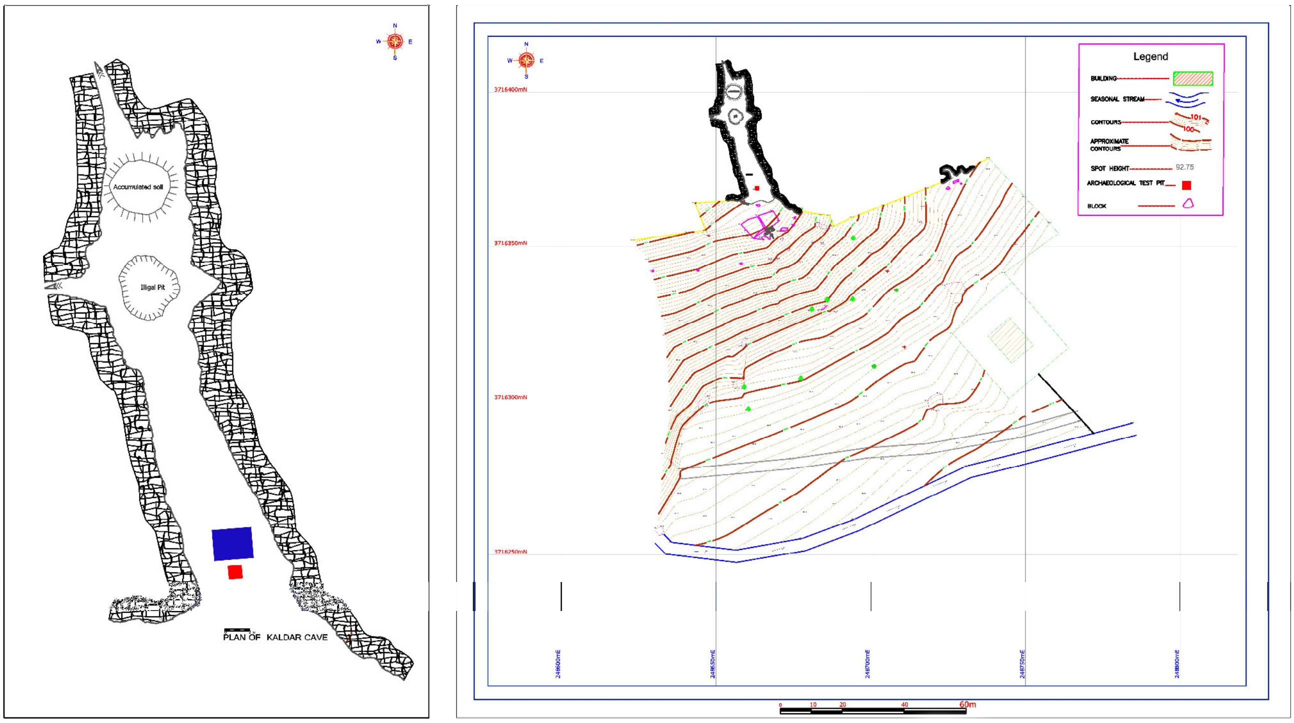

Figure S1. Detailed topographical map of Kaldar and its sounding area. The location of the new trench is shown in blue colour. Created by B. Bazgir, using AutoCad, Photoshop and Corel Draw softwares.

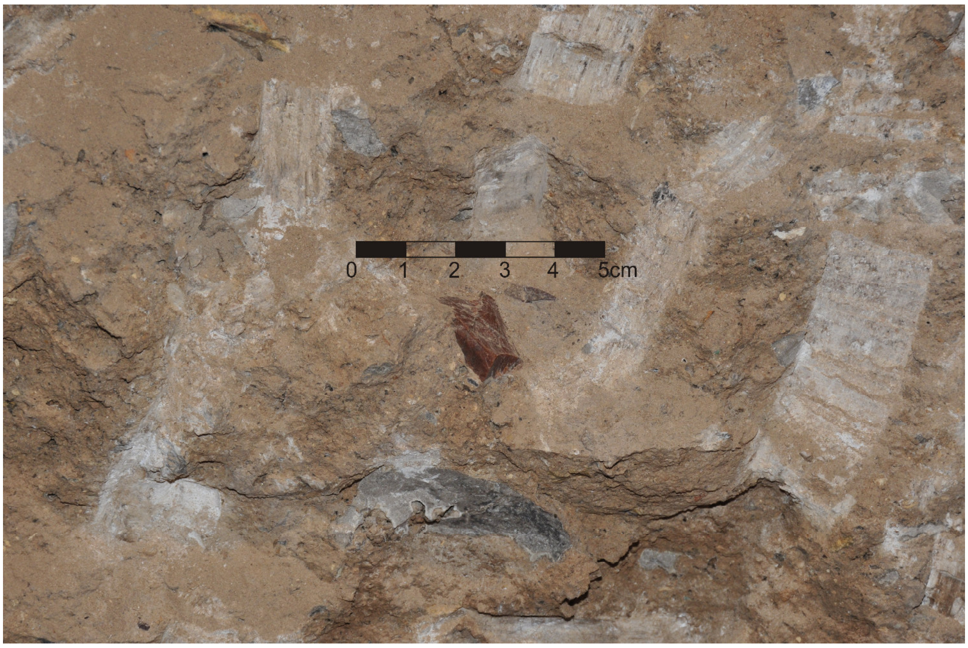

Figure S2. The high density of the deposit can be clearly seen with the traces of chisel marks. Photo by A.Ollé, modified by B.Bazgir.

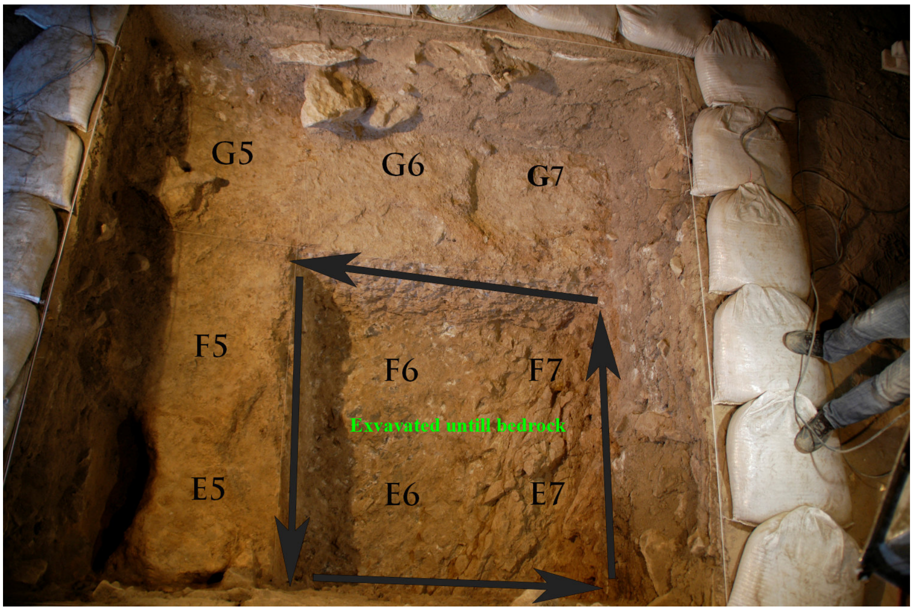

Figure S3. General view of the excavated trench. The squares between the arrows reached to the bedrock. Photo by B. Bazgir.

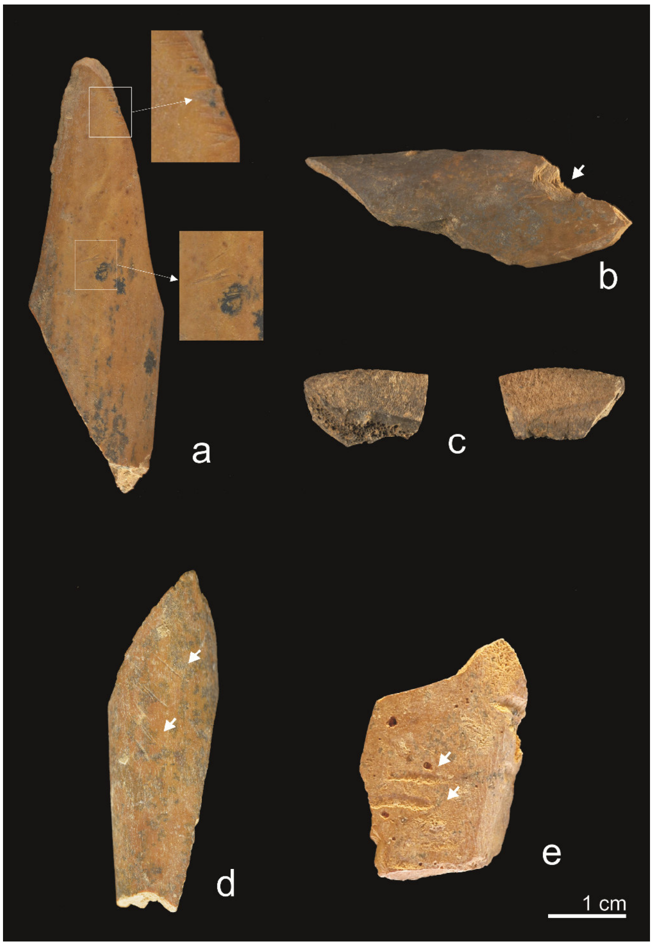

Figure S4. Examples of taphonomic modifications on Kaldar cave fauna specimens. a) Tibia shaft fragment of Cervidae with two groups of slicing marks (Layer 4); b) Percussion impact on the shaft of a Caprini humerus (Layer 4); c) Dorsal and ventral view of burned epiplastron of *Testudo* sp. (Layer 4); d) Slicing marks on a fragment of the shaft of a tibia of Caprini with two groups of slicing marks (Layer 5); e) Fragment of a long bone with carnivore score marks (Layer 4). Created by P. Saladié.

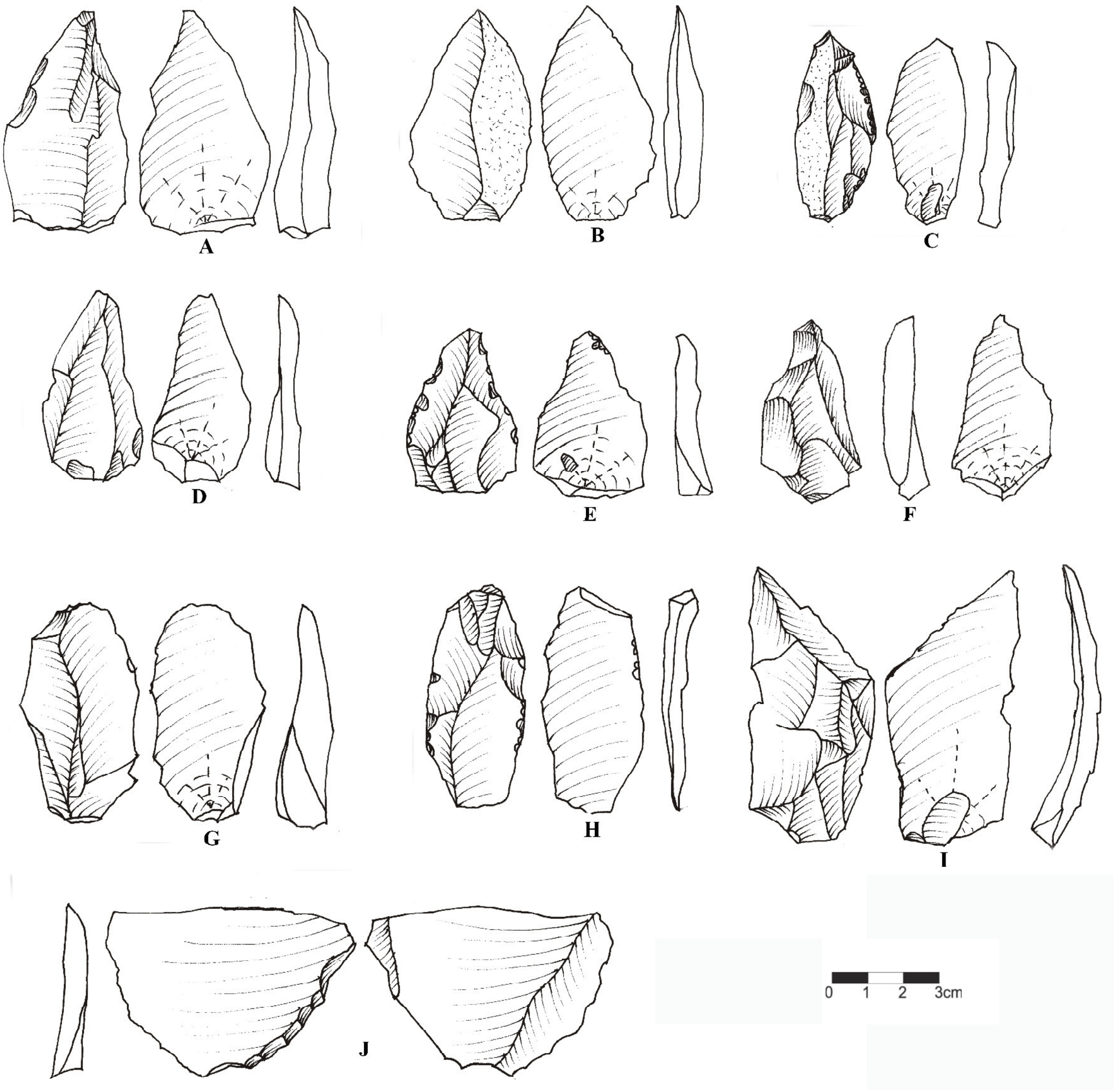

Figure S5. Selection of Levallois pieces from the Middle Paleolithic from Kaldar Cave (Layer 5). A&B; Point, C; Elongated cortical point/pointed flake with cortical butt, D to F; Levallois point, G&H; Elongated Levallois flake, I; Levallois elongated pointed flake, J; Levallois flake. Drawings by L. Tumung.

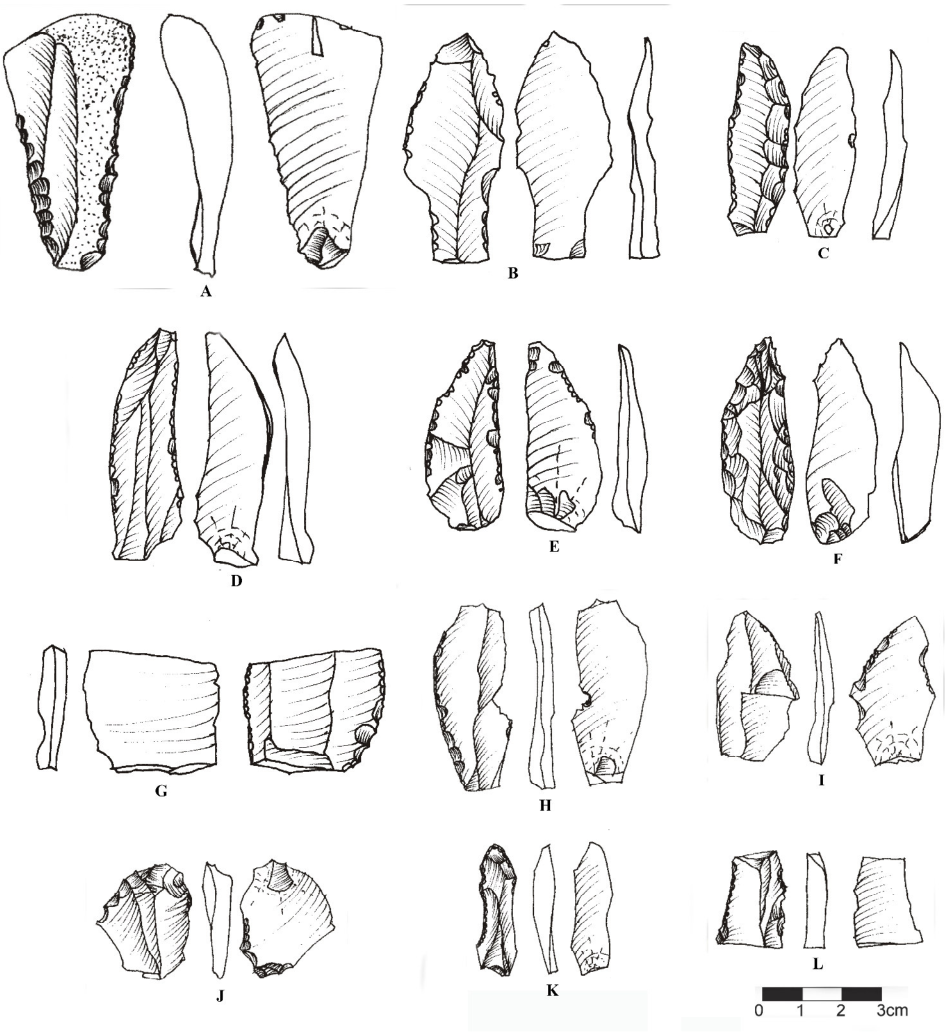

Figure S6. Selection of retouched pieces from the Upper Paleolithic of Kaldar Cave (Layer 4). A; Cortical retouched double scraper, B; Tanged point, C to F; Arjeneh points, G; Retouched blade, H; Elongated retouched blade, I; Point on blade with retouches on the distal portion of the ventral face, J; Retouched end scraper, K; Retouched bladelet point, L; Mesial portion of a retouched bladelet point. Drawings by L. Tumung.

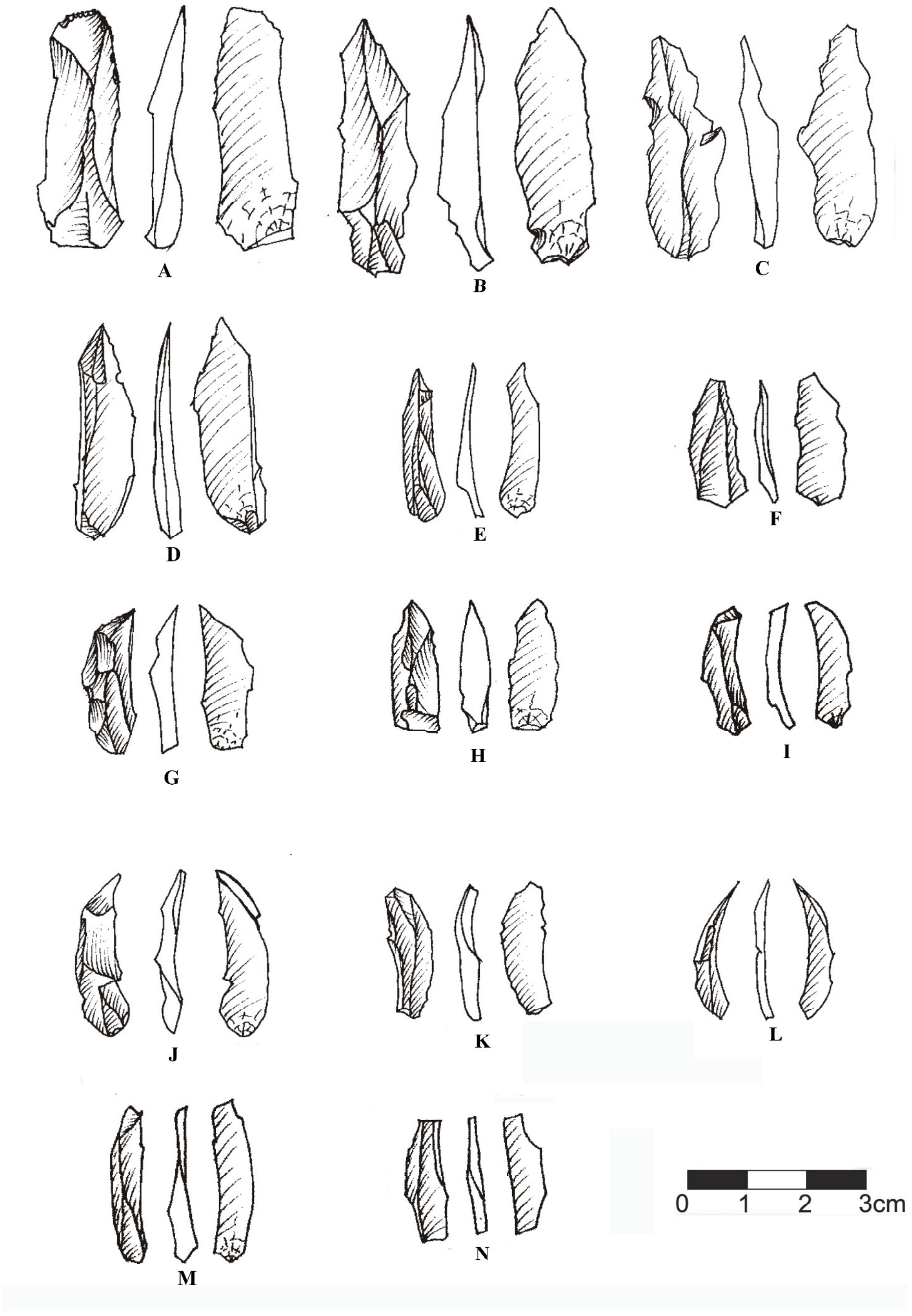

Figure S7. Selection of blades and bladelets of Upper Paleolithic from Kaldar Cave (Layer 4). A; Elongated blade, B to D; elongated pointed blades, E to H; Pointed bladelets, I &N; Dufour bladelets, J to M; Twisted bladelets. Drawings by L. Tumung.

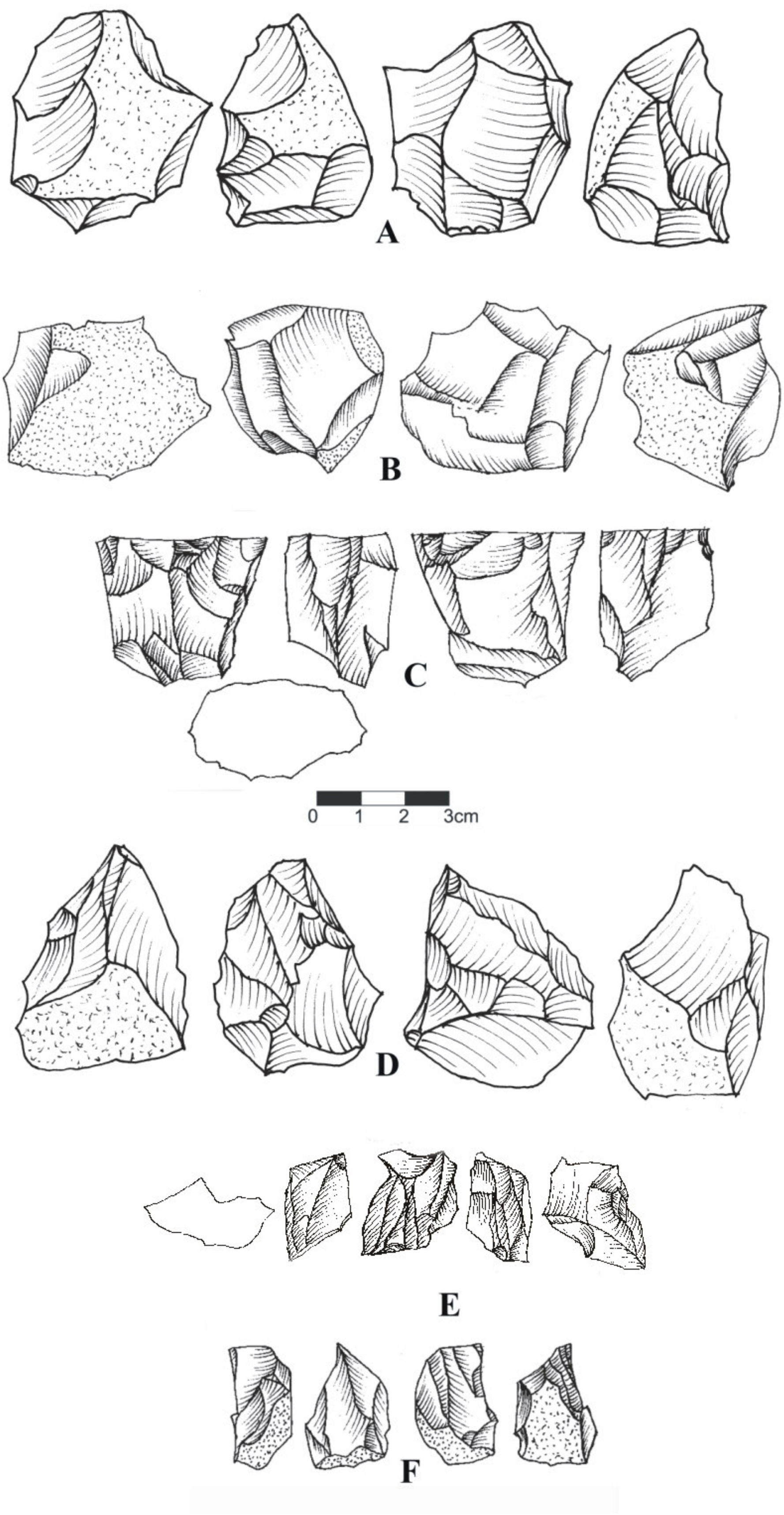

Figure S8. Selection of cores of Upper Paleolithic from Kaldar Cave (Layer 4). A: Blade core, B,C & F; Bladelet core, D; Broken carinated core E; Carinated core/carinated scraper. Drawings by L. Tumung.

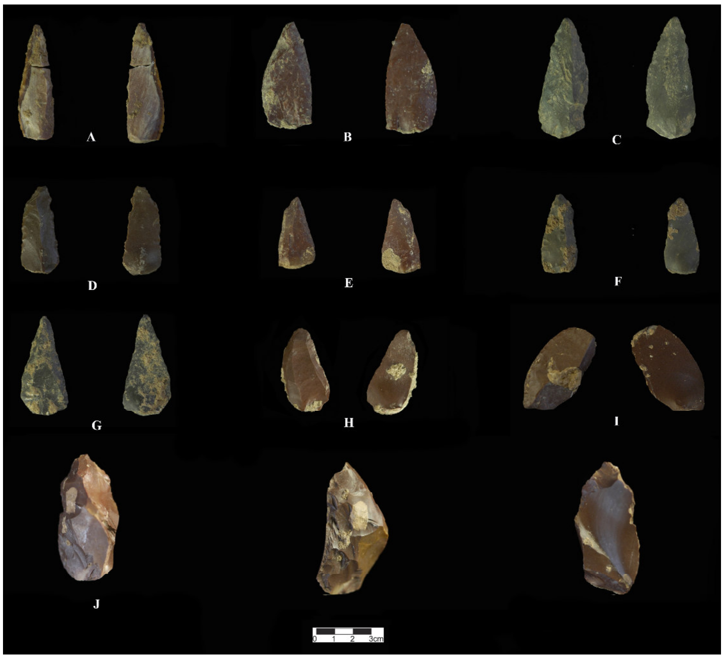

Figure S9A: Selection of retouched pieces from the Middle Paleolithic of Kaldar Cave (Layer 5). A to G; Mousterian points, H&I; Retouched side scrapers, J; Limace. Created by B.Bazgir.

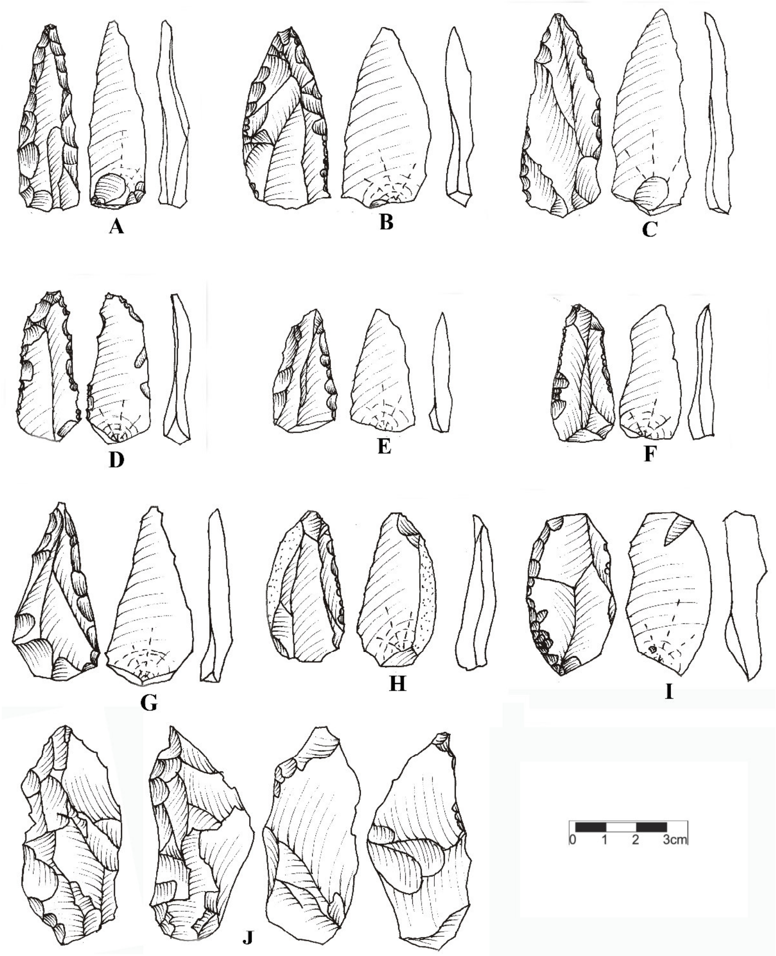

Figure S9B. Selection of retouched pieces of Middle Paleolithic from Kaldar Cave (Layer 5). A to G; Mousterian points, H&I; Retouched side scrapers, J; Limace. Ddrawings by L. Tumung.

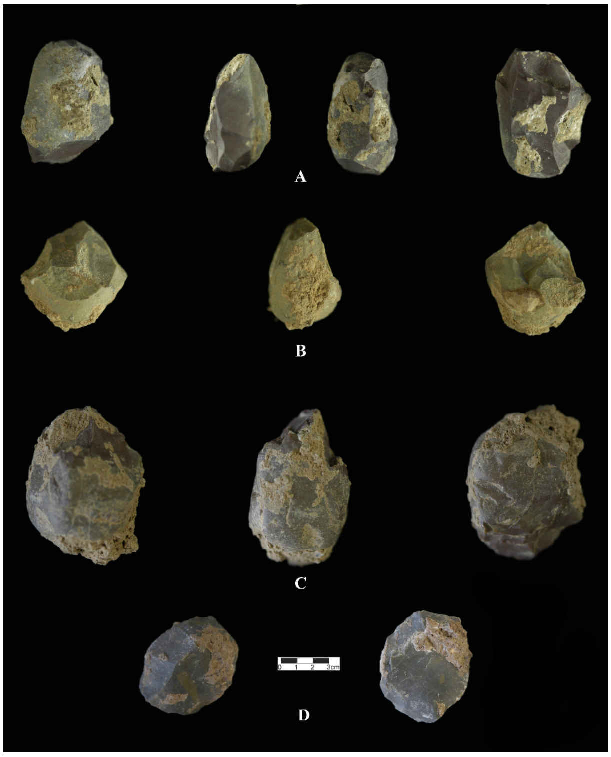

Figure S10A. Selection of cores of Middle Paleolithic from Kaldar Cave (Layer 5). A; Cortical Levallois unidirectional core, B; Cortical unidirectional core C; Predetermining centripetal core, D; Levallois centripetal core. Created by B.Bazgir.

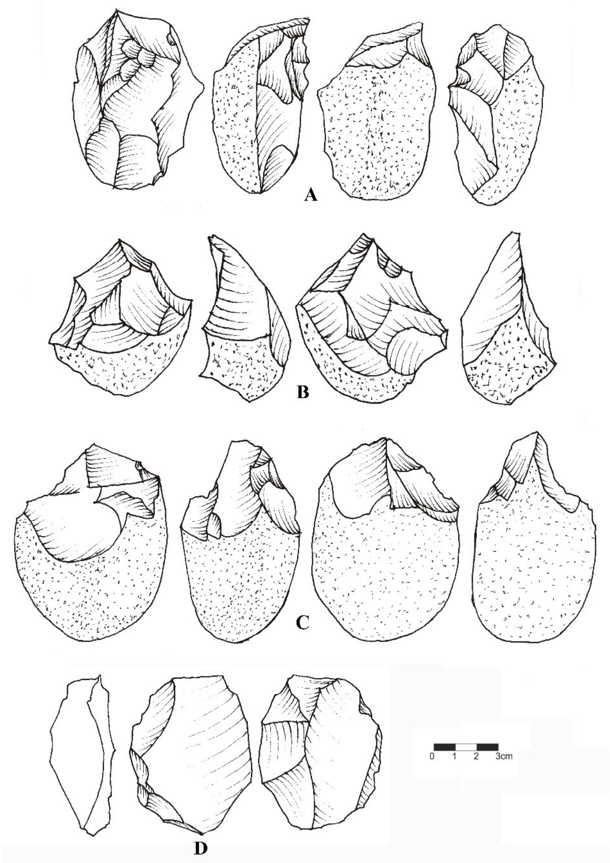

Figure S10B. Selection of cores of Middle Paleolithic from Kaldar Cave (Layer 5). A; Cortical Levallois unidirectional core, B; Cortical unidirectional core C; Predetermining centripetal core, D; Levallois

centripetal core. Drawings by L. Tumung.

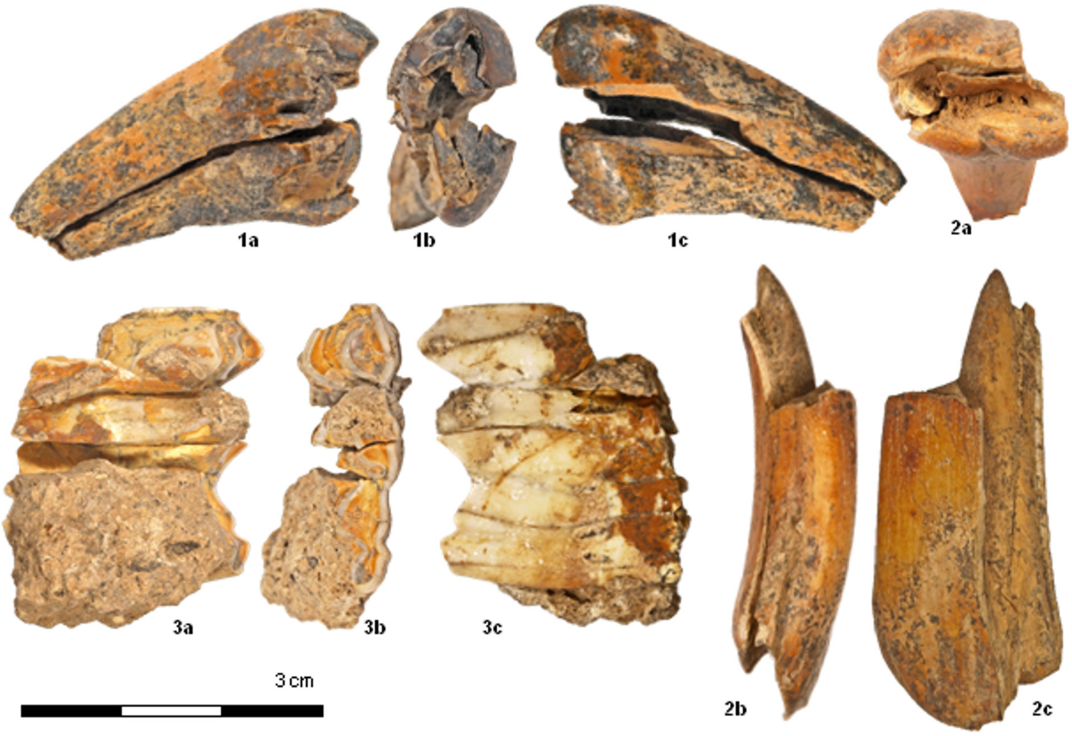

Figure S11. Fossils from Kaldar Cave: 1) KLD-2014- E5-1430 - right upper canine of a female of *Cervus elaphus* from Layer 5: a) lingual, b) occlusal, and c) buccal views; 2) KLD-2014-E6-879 - left upper incisor of *Equus* sp. from Layer 4 (sub-layer 5 II): a) occlusal, b) mesial, and c) labial views; 3) KLD-2014-F6-814 - left lower third molar of *Capra* sp. from Layer 5 (sub-layer 7); Figure 1/2 not to scale. Created and modified by J.van der Made.

Supplementary diagrams

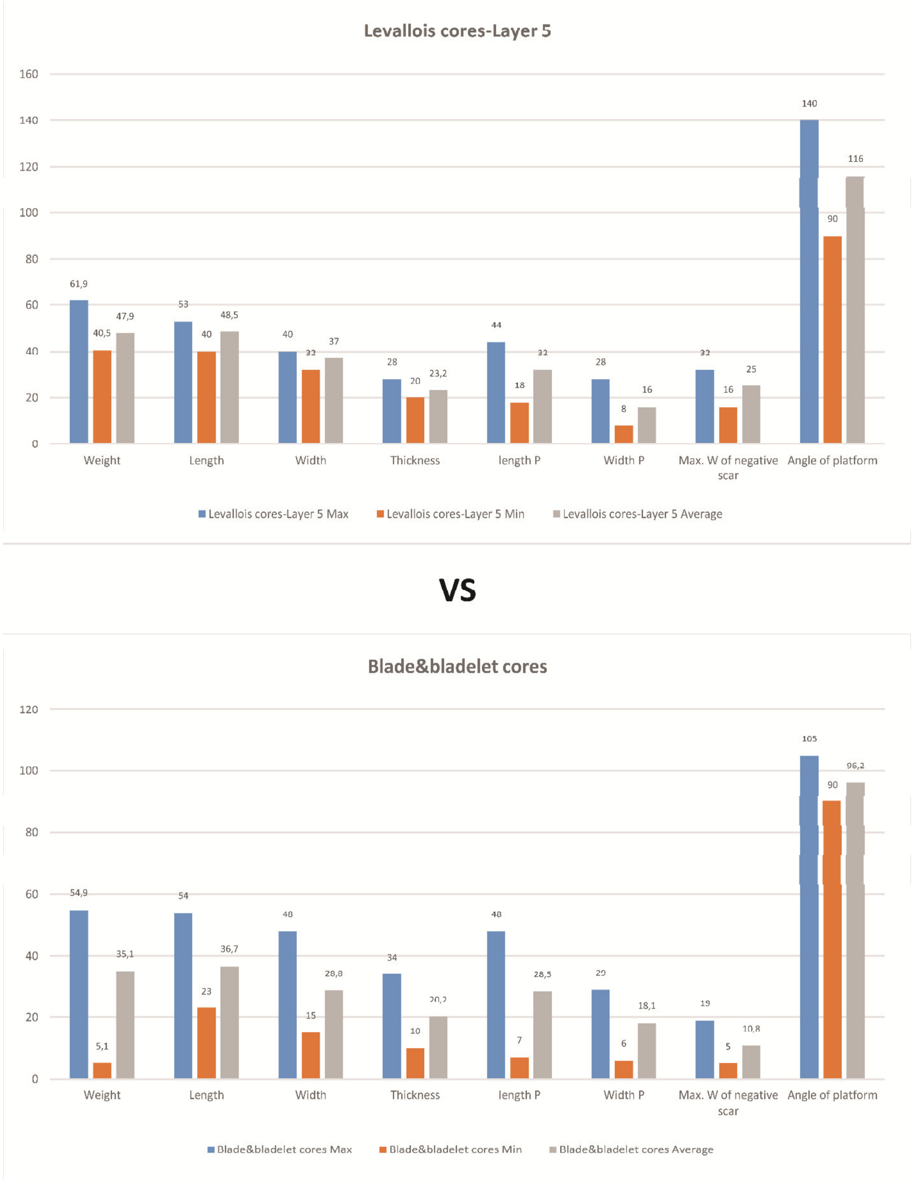

Diagram S1. Comparison of different elements between "Levallois cores vs blade & bladelet cores" within Layers 4 and 5. Created by B.Bazgir.

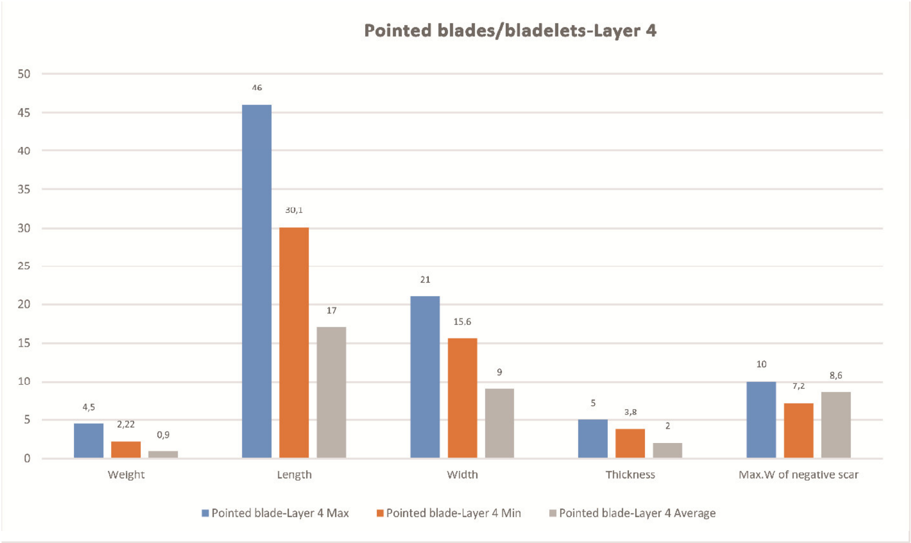

VS

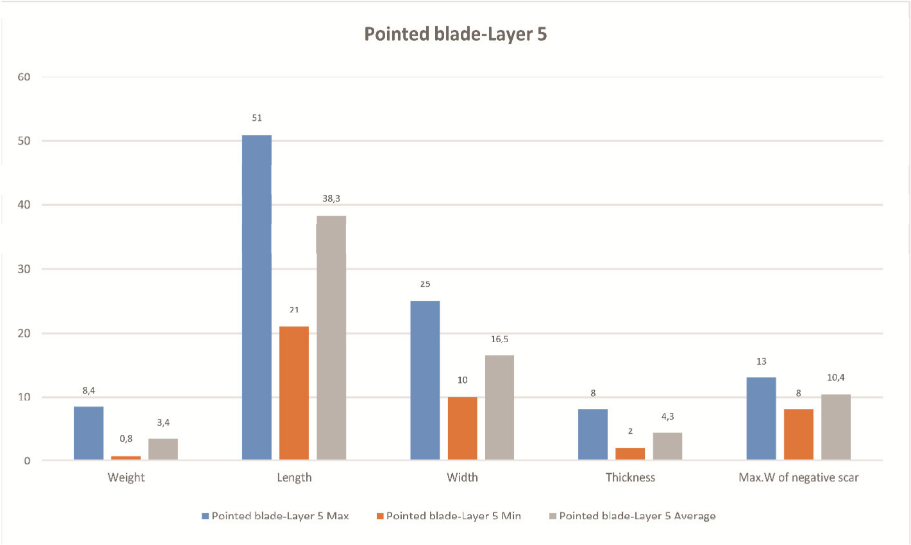

Diagram S2. Comparison of different elements between “retouched points” within Layers 4 and 5. Created by B.Bazgir.

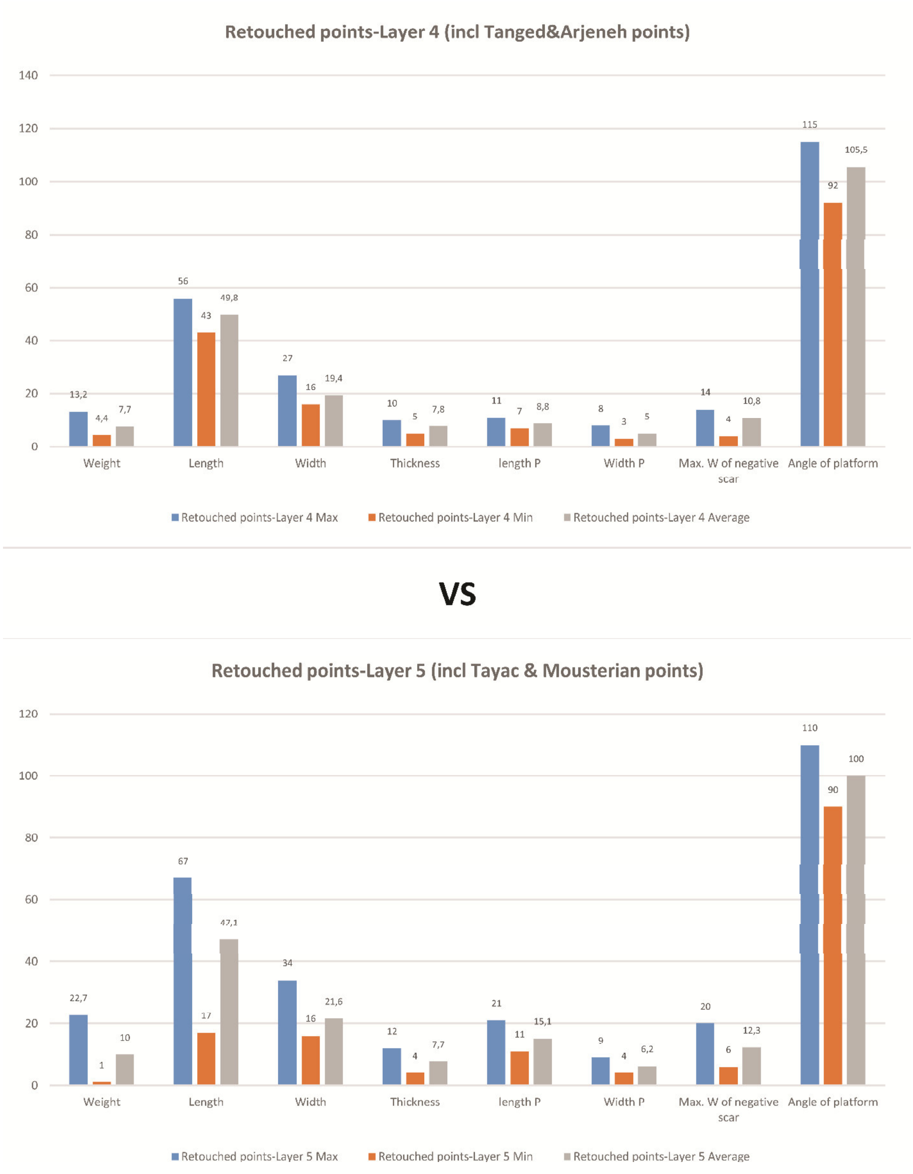

Diagram S3. Comparison of different elements between “pointed blades” within Layers 4 and 5. Created by B.Bazgir.

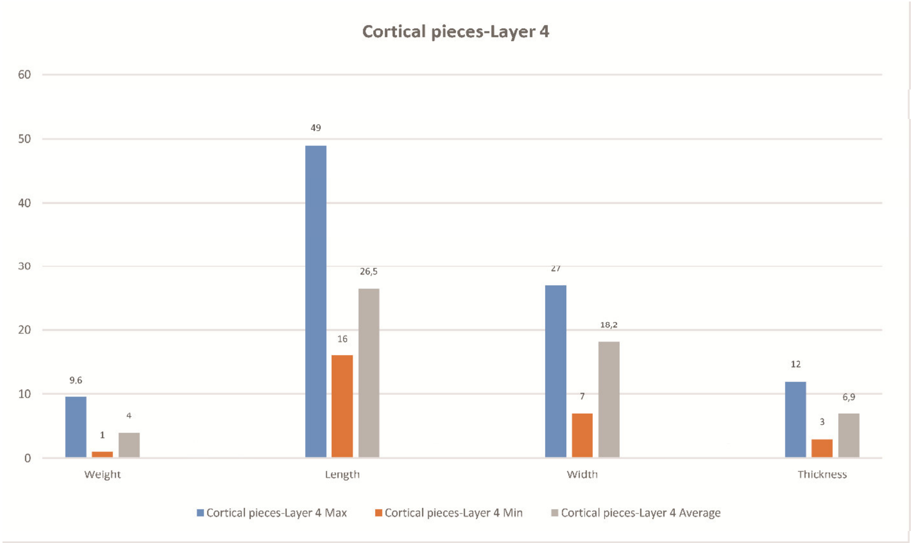

VS

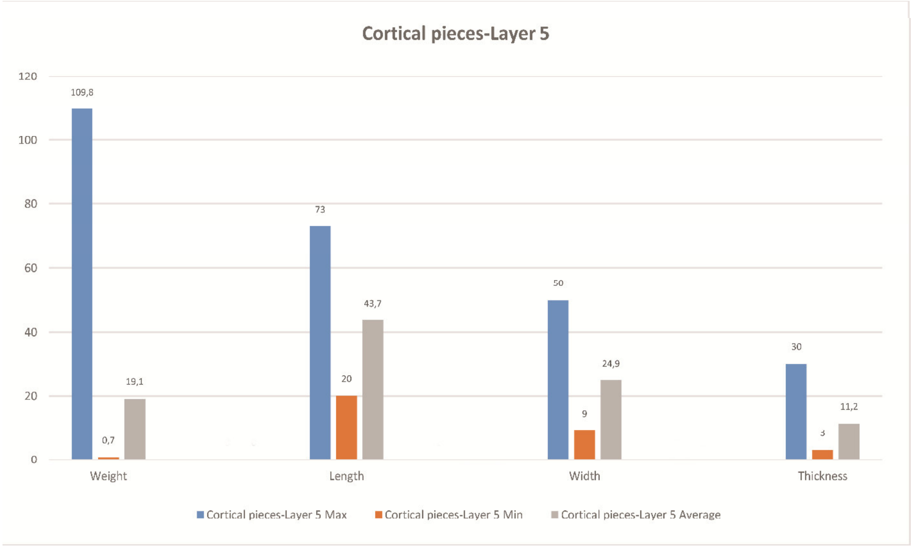

Diagram S4. Comparison of different elements between “cortical pieces” within Layers 4 and 5. Created by B.Bazgir.

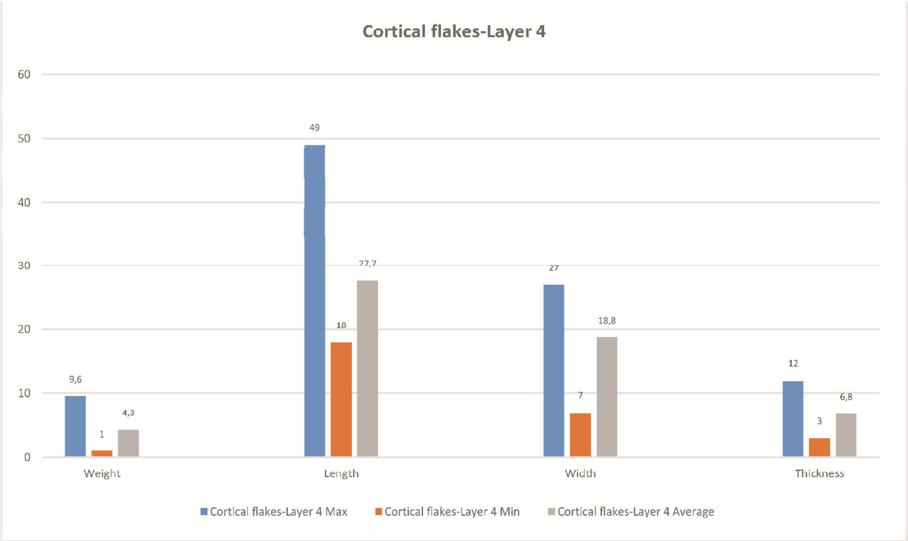

VS

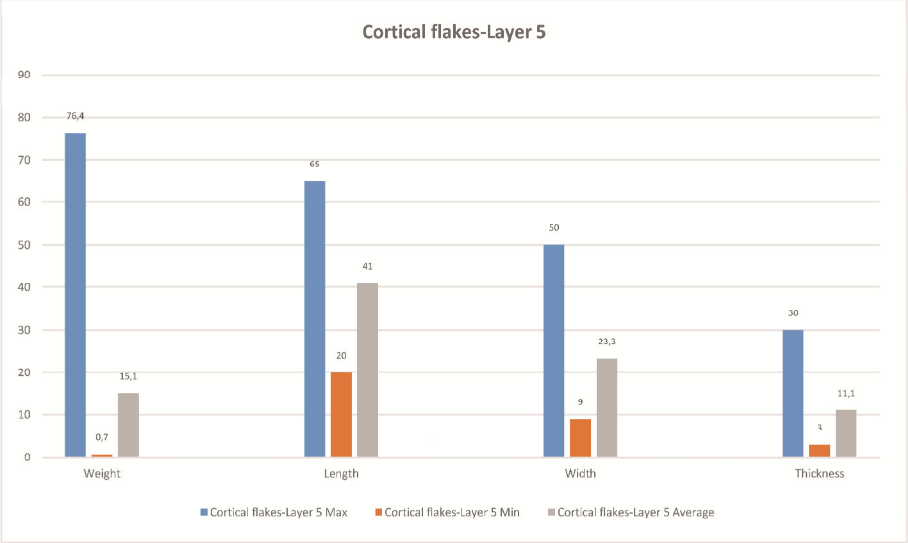

Diagram S5. Comparison of different elements between "cortical flakes" (among the cortical pieces) within Layers 4 and 5. Created by B.Bazgir.
